# Gut microbiome drives glycodeoxycholic acid-mediated attenuation of hypertension

**DOI:** 10.1080/19490976.2026.2691346

**Published:** 2026-06-24

**Authors:** Sachin Aryal, Blair Mell, Ramakumar Tummala, Ishan Manandhar, Sanjana Kumariya, Narendra Kondapalli, Beng San Yeoh, Wisdom Ahlidja, Oluwatosin Mautin Akinola, Sudhan Pachhain, Pritam Bardhan, Piu Saha, Sareh Zeydabadinejad, Islam Osman, Charles Thodeti, Tao Yang, Matam Vijay-Kumar, Lavanya Reddivari, Bina Joe

**Affiliations:** a Center for Hypertension and Precision Medicine, Department of Physiology and Pharmacology, College of Medicine and Life Sciences, University of Toledo, Toledo, OH, USA; b Department of Food Science, Purdue University, West Lafayette, IN, USA

**Keywords:** Secondary bile acids, gut microbiota, blood pressure, TGR5

## Abstract

Gut microbiota and bile acids are increasingly recognized to regulate blood pressure, but the mechanisms remain unclear. Takeda G-protein coupled receptor 5 (TGR5) is a major receptor for secondary bile acids. We hypothesized that loss of TGR5 function remodels gut microbiota and influences blood pressure. Using CRISPR/Cas9, TGR5 knockout (*Tgr5*KO) rats on the Dahl Salt-Sensitive (S) background were generated and characterized. Compared to the control S rats, *Tgr5*KO rats demonstrated significantly lower blood pressure, a distinct shift in gut microbiota composition, and an increase in the secondary bile acid, particularly, glycodeoxycholic acid. Supplementation of glycodeoxycholic acid to the control S rats produced a similar gut microbial shift and lowered blood pressure. Furthermore, cecal microbiota transplantation from *Tgr5*KO to control S rats lowered blood pressure in the recipient rats. This first loss-of-function study demonstrates that deletion of TGR5 remodels gut microbiota, increases glycodeoxycholic acid, and lowers blood pressure regardless of TGR5 signaling status, identifying a promising gut–liver axis target for lowering hypertension.

## Graphical abstract

**Figure:**
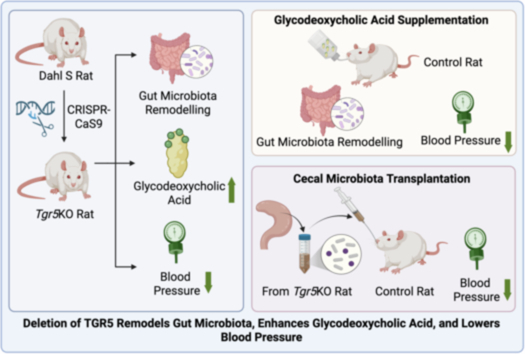

## Introduction

Hypertension or high blood pressure is a major risk factor for cardiovascular diseases. Genetics, environment, and gut microbiota are important factors contributing to the pathogenesis of hypertension.^1–3^ According to the World Health Organization, an estimated 1.28 billion adults globally have hypertension, which has become one of the major reasons for premature death worldwide. Therefore, novel therapeutics are essential to minimize the risk and incidence of hypertension.

In recent years, metabolism of bile acids has emerged as a promising area of investigation for blood pressure regulation.^4^ ^,^ ^5^ Primary bile acids are key metabolites synthesized in the liver from cholesterol, which are converted into secondary bile acids in the intestine by gut microbiota.^6^ Beyond the classical role of bile acids in the emulsification and absorption of dietary fats and liposoluble vitamins, bile acids function as signaling molecules that influence metabolic, immune, and inflammatory processes.^7-9^ We and others have also reported that bile acids play a key role in blood pressure regulation.^4^ ^,^ ^10-12^

Recent findings further highlight the importance of gut–liver axis as an important and previously understudied determinant of cardiovascular health.^13-15^ Within this axis, Takeda G-protein coupled receptor 5 (TGR5), a major receptor for secondary bile acids, has gained attention as a potential novel target for combating obesity, inflammation, and neuropsychiatric disorders.^16-20^ TGR5 is broadly expressed across multiple cell types, including hepatocytes, intestinal epithelial cells, brown adipocytes, skeletal muscle cells, splenic cells, and circulating immune cells, consistent with its role in regulating bile acid signaling, metabolism, and immune responses.^21-23^ Deletion of TGR5 is reported to remodel microbiota composition in a variety of models.^11^ ^,^ ^24^ Furthermore, we and others have demonstrated that microbiota remodeling has a significant effect on blood pressure regulation.^25-30^ These converging lines of evidence prompted us to investigate whether the loss of TGR5 directly or indirectly influences blood pressure through remodeling of the gut microbiota composition.

This is the first loss-of-function study to demonstrate that deletion of the secondary bile acid receptor, TGR5, promotes the abundance of secondary bile acid, glycodeoxycholic acid, remodels the gut microbiome, and lowers blood pressure in Dahl Salt-Sensitive (S) rats. Critically, through three independent and convergent experimental approaches- genetic TGR5 ablation by CRISPR/Cas9, direct glycodeoxycholic acid supplementation, and cecal microbiota transplantation, this study establishes glycodeoxycholic acid as a novel gut‒liver axis-derived antihypertensive metabolite whose blood pressure-lowering effects are observed regardless of TGR5 signaling status, distinguishing our findings from prior studies that attributed bile acid-mediated blood pressure regulation primarily to TGR5 activation. Together, these findings highlight the gut‒liver axis as a key regulator of blood pressure and suggest that enhancing glycodeoxycholic acid levels as an endogenous antihypertensive metabolite could constitute a new strategy to ameliorate hypertension.

## Materials and methods

### Animals and housing conditions

As previously described,^31^ all animal research protocols were reviewed and approved by the Institutional Animal Care and Use Committee of the University of Toledo (Protocol number 104573 for breeding and Protocol number 108390 for experiments). Age-matched female and male wild-type (S) and *Tgr5*KO rats, at least two months old, were used in this study. Animals younger or older than two months, or those showing signs of illness or delayed postsurgical recovery were excluded. The sample size (*n* = 5–9 rats/group/sex) was chosen as a representative subset for the experiments. Rats were randomly assigned to control and experimental groups and housed individually in the Department of Laboratory Animal Resources facility at the University of Toledo. Researchers responsible for data collection and analysis were blinded to group allocation. Animals were housed in cages with *Carefresh* paper bedding, under controlled temperature (70 ± 2 °F), humidity (50 ± 20%), and a 12:12-hour light/dark cycle. Experiments were conducted in accordance with the National Institutes of Health *Guide for the Care and Use of Laboratory Animals*. All experiment procedures and data reporting adhered to the *Animal Research: Reporting of In Vivo Experiments* (ARRIVE) guidelines.

### Generation of TGR5 knockout (*Tgr5*KO) rat model by CRISPR/Cas9 gene editing

*Tgr5*KO rats were generated on the S-rat genetic background using CRISPR/Cas9-mediated gene editing at the University of Michigan Transgenic Animal Model Core (Ann Arbor, Michigan). Briefly, guide RNA was designed to target *Tgr5* locus of exon 2 with no off-target sites. Oocyte microinjections were performed. A mixture of the gRNA and Cas9 mRNA was injected into one-cell stage Dahl S rat embryos. Microinjected embryos were implanted into 8 pseudo-pregnant Dahl S rats. A 1,249 base pair deletion within exon 2 of the *Tgr5* locus on chromosome 9 truncated coding sequence from 2,071 base pairs to 822 base pairs. The founder rat was backcrossed to the S rat, and their pups were intercrossed to obtain homozygosity of the disrupted *Tgr5* allele. Ear tip biopsies of newborn pups were collected, and genomic DNA was extracted. Genotyping was performed using the following primers:

Forward (5′-TTTAGAAGTGAGGGGGTTCTTTGTCCTTA-3′) and

Reverse (5′-CTTTTAGGAGGCCATGTCTCTTTCACTTT-3′). PCR products obtained from the homozygotes after purification were shipped to Eurofins for DNA sequencing. Sequencing analysis confirmed the targeted deletion of TGR5. Rats were bred for five generations to establish a stable line and obtain the required number of homozygous *Tgr5*KO female and male rats.

### Diet

For the entire duration of the study, a low-salt diet (0.3% NaCl, Harlan, Teklad, TD7034) was provided to the rats as previously described.^31^ Both groups were provided with food and water *ad libitum*.

### Food and water intake

Food and water intake were measured every week. Rats were not fasted during the study. At the end of the study, rats were fasted overnight before euthanasia.

### Assessment of energy metabolism

As previously described,^31^ a comprehensive laboratory animal monitoring system (CLAMS, Columbus Instruments) was used for monitoring the energy metabolism. Rats were housed individually for thirty-six hours. Volume of oxygen consumption (VO_2_), volume of carbon dioxide production (VCO_2_), and respiratory exchange ratio (RER) were sampled sequentially for five-seconds in a ten-minute interval for 36 hours. VCO_2_/VO_2_ was used to compute RER. The first twelve-hour readings were excluded as part of the acclimatization of the rats in the CLAMS system and the last twenty-four-hour readings were used for the final calculation. For the entire duration of the study, food and water were provided *ad libitum*.

### Supplementation of glycodeoxycholic acid

The experimental group (*n* = 7–9 rats/group/sex) was supplemented with 100 mg/kg/d of glycodeoxycholic acid in drinking water, whereas the control group was provided with drinking water only.

### Blood pressure measurements

Rats were surgically implanted with radiotelemetry transmitter probes for blood pressure measurement as previously described.^29^ After a week of postimplantation recovery, their blood pressure was monitored. Blood pressure was recorded once every week for 24 hours using the DSI software and equipment (https://www.datasci.com/). Blood pressure (systolic, diastolic, and mean arterial pressure) data were acquired using Dataquest A.R.T 4.36 software. Blood pressure was monitored continuously at 5-minute intervals and analyzed at both 1-hour and 4-hour intervals in all the studies to capture a comprehensive temporal profile of blood pressure changes.

### Blood collection

Before euthanasia, blood was collected from the dorsal aortae into BD microtainer serum-separating tubes (BD Biosciences, Franklin Lakes, NJ). Hemolysis-free sera were collected by centrifugation at 2,000 g for 15 minutes at 4 °C and stored at −80 °C until further analysis.

### Analysis of serum biochemical parameters

As described previously,^31^ total cholesterol (catalog number CH200), alanine transaminase (ALT; catalog number AL146), aspartate transaminase (AST; catalog number AS101), triglyceride (catalog number TR210), and alkaline phosphatase (ALP; catalog number AP313) were measured using the serum samples as per the manufacturer's protocol (Randox). Diazyme assay kit (catalog number DZ042A-KY1) was used to measure total bile acid in serum samples.

### Quantification of urinary total protein

At the end of the study, rats were housed in metabolic cages individually for 24 hours. During this period, access to food was removed, but drinking water was provided *ad libitum*. After 24 hours, the volume of urine excreted was recorded and total urinary protein was quantified using the pyrogallol-based QuanTtest Red Total Protein Assay (Quantimetrix, Redondo Beach, CA, USA) as described previously.^31^ Protein concentration was determined spectrophotometrically at 600 nm using QuanTtest human protein standards (range 25–200 mg/dL) and expressed as total protein excretion (mg/dL of urine in 24 hours).

### Thoracic echocardiography

Cardiac function was assessed using a Vevo2100 system (Visual Sonics Inc., Toronto Canada) with a MX250 transducer (13–24 MHz). Rats were anesthetized with 3% isoflurane and fixed in a supine position on a heat pad at 37 °C (FUJIFILM Visual Sonics, Toronto, Ontario, Canada). Isoflurane concentrations were further reduced to a minimum of 1–2% to achieve constant and comparable heart rates during image acquisition. 2D images were acquired in para-sternal short, long axis at mid papillary level, and apical four-chamber views were obtained. All measurements were calculated offline by a blinded reviewer using Vevo 5.6.0 software. Ejection fraction and fractional shortening were calculated using the formulas (LVEDV-LVESV)/LVEDV and (LVID, d-LVID, s)/LVI. Stroke volume was calculated as: (Diastolic—systolic endocardiac volume). Cardiac output was calculated as: (stroke volume × heart rate).

### Isometric wire myography

Isometric wire myography was performed as discussed in previous publications,^32^ ^,^ ^33^ with some modifications. Aortic segments, along with third- and fourth-order mesenteric resistance arteries (MRA) were harvested after euthanizing the rats. Ice-cold oxygenated modified Krebs bicarbonate (Krebs) buffer (130 mM NaCl, 4.7 mM KCl, 1.17 mM MgSO4, 1.18 mM KH_2_PO4, 1.6 mM CaCl_2_, 25 mM NaHCO_3_, 5.5 mM glucose, and 0.03 mM EDTA; pH 7.4) was used to clean the periadventitial fat and connective tissues from the harvested aortic and MRA segments. A gas mixture of 95% O_2_ and 5% CO_2_ was continuously passed through the Krebs buffer for aeration. Multi-chamber Danish Myo Technology pin was used to mount aortic segments and wire myographs (DMT 620 M) were used to mount MRAs (approximately 2 mm) in the presence of oxygenated Krebs buffer (37 °C). MRAs were stretched steadily to a variable optimal pretension, which was established using a standard DMT normalization process with an internal circumference (IC)1/IC100 of 0.9 and a target pressure of 13.3 kPa. Conversely, aortic segments were stretched to a prefixed tension of 10 mN following an initial stabilization phase. Vessels were incubated for 45 minutes with several wash processes using fresh oxygenated Krebs buffer at 37 °C after achieving a stable pretension. A high-KCl solution (120 mM), cumulative concentrations of the vasoconstrictors phenylephrine (PE) or 5-hydroxytryptamine (5-HT) (10^−9^ − 10^−4^ M), cumulative concentrations of an endothelial-dependent vasodilator, acetylcholine (ACh) (10^−9^ − 10^−4^ M), or an endothelial-independent vasodilator, sodium nitroprusside (SNP) (10^−9^ − 10^−4^ M) were used to challenge endothelium-intact aortic rings or MRAs for testing vascular reactivity. To restore a stable passive basal tension, vessels were repeatedly washed using fresh 37 °C oxygenated Krebs buffer in between different drug treatments. Following initial preconstriction, relaxation experiments were performed using a submaximal concentration of PE (10^−7^ M for aortic rings or 10^−6^ M for MRA). The effects of cumulative PE doses were repeated in certain experiments with L-NAME (100 mM, 30 minutes pretreatment), a nitric oxide synthase inhibitor.

### Fecal pellet collection

Fecal pellets were aseptically collected from individual rats at the end of the study and immediately stored at −80 °C for subsequent 16S rRNA gene sequencing and whole-genome sequencing analyzes.

### DNA isolation

As discussed previously,^34^ ^,^ ^35^ gDNA was extracted from the fecal pellets (approximately 40–50 mg) of S rats, *Tgr5*KO rats, GDCA-supplemented S rats, and control S rats using QIAamp Power Fecal Pro DNA Kit (QIAGEN) as per their protocol. The gDNA was eluted in low-TE buffer (0.1 mM EDTA, Tris-HCl buffer, 10 mM, pH 8.5). A NanoDrop was used to measure DNA concentration. For 16 s ribosomal RNA sequencing (16S rRNA), DNA was diluted to a final concentration of 5 ng/μl in low-TE buffer. For whole genome sequencing by GridION from Nanopore, the genomic DNA was isolated using wide-bore tips to avoid undue fragmentation of the DNA. Agarose gel electrophoresis was performed to assess the quality of the DNA.

## 16S rRNA gene sequencing and analysis of microbiota composition

### 16S polymerase chain reaction library preparation, clean-up, normalization, and pooling

The 16S rRNA gene library preparation, clean-up, normalization, and pooling were performed as previously described.^34-36^ We followed the Illumina *16S Metagenomic Sequencing Library Preparation Guide* - *Preparing 16S Ribosomal RNA Gene Amplicons for the Illumina MiSeq System* (Part No. 15044223 Rev. B). The V3-V4 hypervariable region of the 16S rRNA gene was amplified by PCR using Illumina adapter-linked primers:

5′ TCGTCGGCAGCGTCAGATGTGTATAAGAGACAGCCTACGGGNGGCWGCAG and 5′ TCTCGTGGGCTCGGAGATGTGTATAAGAGACAGGGACTACHVGGGTWTCTAAT. For index PCR, dual indexes were attached using the Nextera XT index kit (FC-131-1002, Illumina). Each 25 µL reaction mixture contained 2.5 µL of 10X reaction buffer (Invitrogen, Thermo Fisher Scientific, Waltham, MA), 0.5 µL of 10 mM dNTPs, 0.75 (for target PCR)/1 µL (for index PCR) of 50 mM MgCl2, 0.1 µL of 5U/µL of HotTaq polymerase (Invitrogen), 1 µL of each primer (5 µM) and 2.5 µL of 5 ng/µL DNA template. The reaction volume was adjusted to 25 µL with nuclease-free water. A BioRad T100TM thermal cycler (Hercules, CA, USA) was used for thermocycling. Initial denaturation at 95 °C for 5 minutes, 25 cycles of 95 °C for 30 seconds, 58 °C for 30 seconds, 72 °C for 30 seconds, and a final extension at 72 °C for 5 minutes were used as the target PCR conditions. Initial denaturation was conducted at 95 °C for 3 minutes, followed by 95 °C for 30 seconds, 55 °C for 30 s, 72 °C for 30 seconds, and a final extension at 72 °C for 5 minutes for the index PCR for 8 cycles. PCR amplicons were purified twice using AMPure XP beads (Beckman Coulter Inc. Brea, CA, USA). DNA concentration of purified index PCR products was quantified using the Qubit dsDNA HS Assay kit with Qubit 3.0 fluorometer (Life Technologies, Carlsbad, CA, USA). An equimolar amount of 4 nmol/L of each amplicon was pooled, and library quality was assessed using an Agilent 2100 Bioanalyzer (Santa Clara, CA, USA). Library denaturation and MiSeq sample loading were performed according to the Illumina *MiSeq System User Guide*. The final library (10 pmol/L) containing 10% PhiX was loaded onto an Illumina MiSeq V3 flow cell with 2 × 300 cycles for sequencing.

## Quality filtering, amplicon sequencing variant picking, and data analysis

Chimeric sequences were detected and filtered using Quantitative Insights in Microbial Ecology version 2 (QIIME 2) (2021.11). After that, the amplicon sequence variants (ASVs) were subsequently identified within QIIME 2. Taxonomy was assigned to ASVs using a pre-trained Naïve Bayes classifier. Silva V132, clustered at 97% identity, was used as the taxonomic reference database. Bray–Curtis, Jensen-Shannon Divergence, and Jaccard index principal coordinate analysis (PCoA) performed using the previously reported method, Microbiome Analyst^37^ utilizing the taxonomy abundance file generated from QIIME2 analysis.

## Nanopore GridION metagenomics sequencing of fecal samples

Native Barcoding Kit 24 V14 SQK-NBD114.24 was used and ligation sequencing kit protocol (Nanopore) was followed.

### DNA repair and end-preparation

For each sample, 1 μg of DNA (in 11 μl volume) was combined with 1 μl DNA control strand, 0.875 μl l NEBNext FFPE DNA repair buffer, 0.875 μl NEBNext FFPE DNA repair mix, 0.875 μl ultra II end-prep reaction buffer, 0.75 μl Ultra II End-prep Enzyme mix and 0.5 μl NEBnext FFPE DNA Repair Mix was added in 0.2 ml thin walled PCR tubes. The components were thoroughly mixed by pipetting and spun down. The mixture was incubated in a thermal cycler at 20 °C for 5 minutes followed by 65 °C for 5  minutes. Next, 15 μl of resuspended AMPure XP Beads (AXP) were added to the end-prep reaction in Eppendorf DNA in LoBind tubes. After a 5-minute incubation on a (Hula mixer) at room temperature, the tubes were placed on a magnetic rack to pellet the beads. The clear supernatant was carefully removed, and the pellet was washed twice with 200 μl of freshly prepared 80% ethanol. After ensuring no residual ethanol remained after airdrying for 30  seconds, the pellet was resuspended in 10 μl of nuclease-free water. Following magnetic separation of the beads, the 10 μl elute was collected. Finally, 1 μl of the eluted sample was quantified using a Qubit fluorometer to determine the DNA concentration.

### Native barcode ligation

In a 0.2 ml PCR tube, 20 fmol of each end-prepped DNA in a 7.5 μl volume was added, followed by 2.5 μl Native barcode (NB01-24) and 10 μl Blunt/TA Ligase master mix. The reaction was thoroughly mixed and spun down, followed by incubation for 20 minutes at RT. Two microliters of EDTA supplied in the kit were added to stop the reaction. All the bar-coded samples were pooled and purified by the addition of 0.4X AMPure XP Beads, mixed by pipetting, and incubated on a rotator mixer for 10 minutes at RT. The pooled barcoded samples were spun down and pelleted on a magnet. The beads were washed twice with 700 μl of freshly prepared 80% ethanol without disturbing the pellet. The residual ethanol was pipetted off and air dried for 30 seconds. This was followed by the addition of 35 μl Nuclease-free water and incubating at 37 °C for 10 minutes followed by elution a magnetic rack. A total of 1 μl of eluted sample was quantified by qubit fluorimeter.

### Adapter ligation and clean-up

Thirty microliters of pooled barcoded samples was combined with 5 μl of Native Adapter (NA) and 10 μl of NEBNext quick Ligation reaction buffer (5×) followed by the addition of 5 μl Quick T4DNA Ligase. in a 1.5 ml Eppendorf LoBind tube. The mixture was incubated at room temperature for 20 minutes to allow ligation. Following ligation, 20 μl of resuspended AMPure beads were added. After a 10-minute incubation on a rotator mixer at room temperature, the tubes were placed on a magnetic rack to pellet the beads. The supernatant was removed, and the beads were washed twice with 125 μl Long Fragment Buffer (LFB). After the washes, the beads were pelleted again on the magnetic stand and resuspended in 15 μl elution buffer. The solution was incubated at 37 °C for 10  minutes with gentile agitation every 2 minutes. then placed back on the magnet to collect eluate, which contained the DNA library. The DNA concentration of the library was measured using a Qubit fluorometer, and the final library was prepared at 12 μl with −20 fmol of DNA library for sequencing.

### Priming and loading the prepared library onto the SpotON flow cell

The flow cell priming mix was prepared by making a mixture of 1170 μl of Flow Cell Flush buffer (FCF), 5 μl of bovine serum albumin at 50 mg/ml followed by the addition of 30 μl of Flow cell Tether (FCT). The priming of the flow cell (10.4.1) was done as per the instructions from the Nanopore Technical brochure.

### Loading the library

In total, 12 μl of the DNA library was combined with 37.5 μl of sequencing buffer and 25.5 μl of library solution and then loaded on the SpotON flow cell (10.4.1) onto the GridION according to instructions provided in SQK-NBD114.24 protocol. Sequencing was performed on a GridION device with high-accuracy base calling enabled for 72 hours.

### Microbiome data analysis

The passed FASTQ files were exported and processed through the CZ-ID (Chan Zuckerberg Initiative) portal, where host and human reads were first filtered out. The barcodes and adapters were removed utilizing the Dorado pipeline. The reads were then processed with Biobam Software solutions, which encompasses OmicsBox with a metagenomic module. The resulting species-level read counts were normalized to 1 million sequenced bases and visualized utilizing the differential abundance module, which generates a heat map of differences in species abundance with an FDR-adjusted *p*-value < 0.05.

## Targeted bile acid quantification

For bile acid quantification in *Tgr5*KO and control S rats, serum samples were collected and shipped to the West Coast Metabolomics Center at the University of California, Davis (https://metabolomics.ucdavis.edu/) for targeted bile acid profiling. Briefly, samples were processed using the AbsoluteIDQ Bile acids kit (Biocrates). Samples were injected using a Thermo Vanquish Horizon UHPLC with a Thermo Altis system for data acquisition. Data processing was done with the Biocrates online software. For bile acid quantification in the glycodeoxycholic acid supplementation study and cecal microbiota transplantation study, serum samples were collected and shipped to Purdue University. Bile acids were extracted from serum samples as previously described.^38^ Briefly, 150 µL of ice-cold methanol containing 0.4 µg/mL of deuterated chenodeoxycholic acid, as an internal standard (CDCA-d), was combined with 50 µL of serum. The mixture was centrifuged at 4 °C using 14,000 × g for 20 minutes after being kept at −20 °C for 20 minutes. Analysis was done using the supernatant. Quantification of targeted conjugated and unconjugated bile acids was done using an Agilent 1260 Infinity II HPLC system coupled with an Ultivo triple quadrupole mass spectrometer.^39^ To enhance sensitivity for bile acid quantification, the analytical method was carried out in negative multiple reaction monitoring mode. Solvent A (20 mM ammonium acetate with 0.1% (v/v) formic acid) and solvent B (methanol) were combined to create the mobile phase. A Supelco Ascentis Express C18 column (150 × 4.6 mm, 2.7 μm) kept at 40 °C was used to separate the analytes. Gas temperature 200 °C, gas flow 12 L/minutes, nebulizer pressure 40 psi, sheath gas temperature 200 °C, sheath gas flow 10 L/minutes, and capillary voltage 3,000 V were the specifications of the mass spectrometer. For each bile acid, standard curves were used for quantification.

## Tissue collection

At the end of the studies, after the blood collection, all rats were euthanized under surgical isoflurane anesthesia by cardiectomy. Tissues were harvested, flash frozen using liquid nitrogen, and stored at −80 °C until further processing.

## Reverse transcription-polymerase chain reaction

Total RNA was extracted from the ileum of rats using the TRIzol reagent following the method previously described.^40^ RNA concentration and purity were assessed using a NanoDrop spectrophotometer (Thermo Scientific, Serial 1998). Complementary DNA (cDNA) was synthesized from the extracted RNA using the SuperScript III First-Strand Synthesis System (Invitrogen). Quantitative real-time PCR (qRT‒PCR) was performed using SYBR Green Master Mix (Applied Biosystems) and primers specific for the Takeda G-protein–coupled receptor 5 (*Tgr5*). Gene expression levels were normalized to *β-Actin* and calculated using the 2^–∆∆Ct^ method.^41^ Primer sequences used in this study are provided in Supplemental Table S1.

## Cecal microbiota transplantation

Donor S rats and donor *Tgr5*KO rats (*n* = 6/group/sex) were euthanized, and their cecal contents were collected separately, stored on ice in sterile containers, and processed immediately under anaerobic conditions. A separate group of male and female S rats (*n* = 14–16 rats/sex) was assigned into two groups for each sex: Group 1 receiving cecal microbiota from donor S rats and Group 2 receiving cecal microbiota from donor *Tgr5*KO rats. The collected cecal content was weighed and mixed with three volumes of sterile phosphate-buffered saline (PBS). The resulting slurry was homogenized and centrifuged at low speed (520 rpm, 2 minutes) at 4 °C. Following separation of undigested feed and particulate material from the microbial fraction, the supernatant containing the microbial fraction was aliquoted into Hungate tubes. Sterile 20% glycerol was added anaerobically in a 1:1 ratio to each aliquot, and the samples were stored at −80 °C for subsequent use. Recipient rats were orally gavaged with 500 µl of the cecal microbiota suspension for three consecutive days, followed by weekly doses thereafter for up to 4 weeks. Blood pressure was monitored weekly after the initiation of microbiota transplantation. Prior to the transplantation, all rats were pretreated with antibiotics (vancomycin and meropenem; each 50 mg/kg/d) administered in drinking water and omeprazole (50 mg/kg/d) delivered by oral gavage for 3 d (Figure 5A) to deplete resident gut microbiota.

## Statistical analyzes

All statistical analyzes were performed using GraphPad Prism version 10.3.1 for Windows (GraphPad Software, Boston, MA, USA; www.graphpad.com). Data from female and male rats were analyzed separately, and comparisons between control and experimental groups were conducted independently for each sex. Differences between the two groups were assessed using an unpaired, two-tailed Student's *t*-test with a 95% confidence interval. For blood pressure measurements, repeated-measures 2-way ANOVA followed by Tukey multiple comparison test was used as the primary analysis to compare data between the groups at each time point. An ordinary 2-way ANOVA was used to assess the overall difference between the two groups. CLAMS readings were analyzed using 2-way ANOVA followed by Fisher's least significant difference (LSD) test. Data are presented as mean ± SEM, and statistical significance is reported as p*-values.* Outlier calculator (GraphPad Prism 10) was used to identify outliers with *α* = 0.05 and excluded from graphing and statistical analyzes.

## Results

### Genetic ablation of TGR5 attenuated blood pressure independent of hepatic, renal, cardiac, and vascular functions

**Figure 1.**
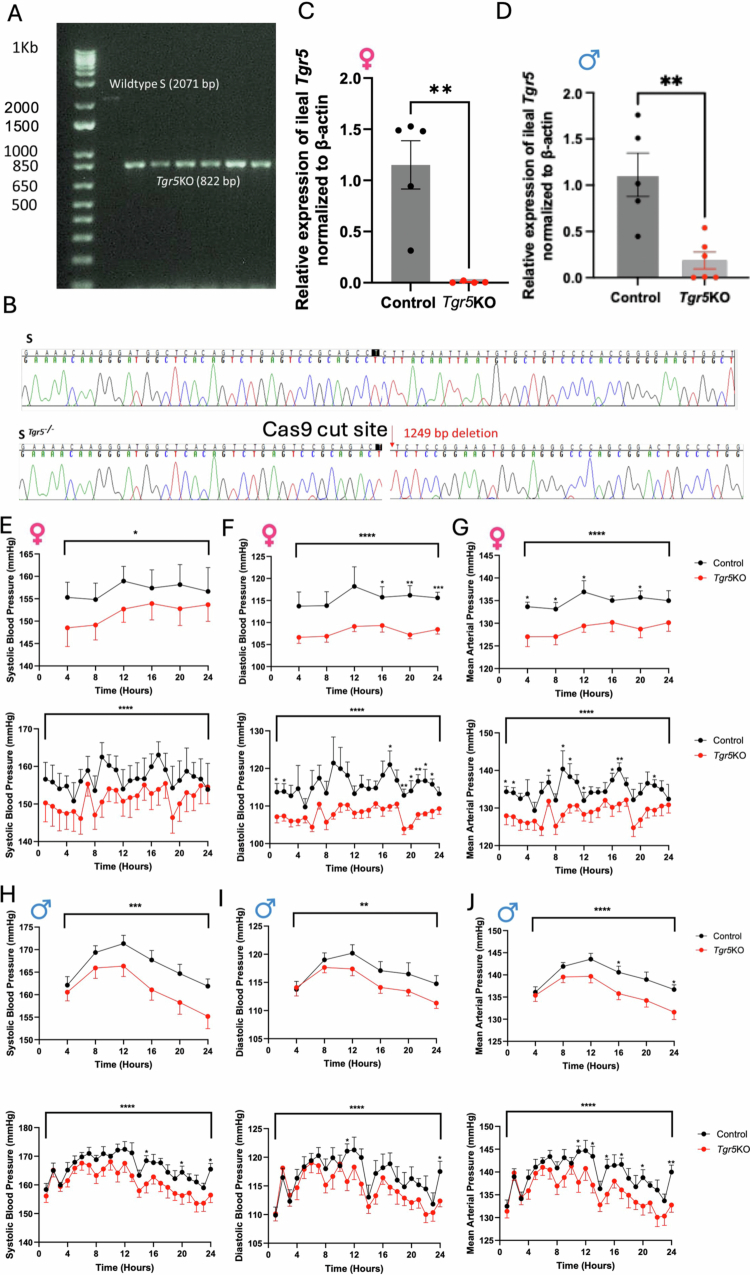
Screening and characterization of rats for CRISPR/Cas9-mediated deletion of *Tgr5* locus and the lowering of blood pressure. (A) Genotyping data. (B) Sequencing result. (C-D) Relative expression of *Tgr5* in ileum of female (C) and male (D) rats. 24-hour blood pressure data of female rats (E) Systolic (Top panel: 4-hour averages, Bottom panel: 1-hour averages). (F) Diastolic (Top panel: 4-hour averages, Bottom panel: 1-hour averages). (G) Mean arterial pressure (Top panel: 4-hour averages, Bottom panel: 1-hour averages). 24-hour blood pressure data of male rats (H) Systolic (Top panel: 4-hour averages, Bottom panel: 1-hour averages). (I) Diastolic (Top panel: 4-hour averages, Bottom panel: 1-hour averages). (J) Mean arterial pressure (Top panel: 4-hour averages, Bottom panel: 1-hour averages). *Tgr5*KO: Takeda G-protein coupled receptor 5 knock out. *n* = 4-7 rats/group/sex. For blood pressure measurements, repeated measures 2-way ANOVA followed by Tukey multiple comparison test was used as the primary analysis to compare data between the groups at each time point. An ordinary 2-way ANOVA was used to assess the overall difference between the two groups. An unpaired, parametric two-tailed Student's *t* test with a 95% confidence interval was used to compare data between groups (Figure 1C and D). All data are mean ± SEM, *p < 0.05, **p < 0.01, ***p < 0.001, ****p < 0.0001.

Exon 2 of the *Tgr5* gene in the S rat was targeted for editing using the CRISPR/Cas9 technology. This resulted in a 1249 base pairs deletion, which could be tracked as 822 base pairs product representing the homozygous alleles for *Tgr5*KO (Figure 1A and B). We confirmed that the relative expression of *Tgr5* in the ileum was significantly reduced in both female and male *Tgr5*KO rats compared to their respective controls (Figure 1C and D). Blood pressure recordings after 4 weeks of radiotelemetry surgery showed a significant reduction in 24-hour systolic, diastolic, and mean arterial pressures in both *Tgr5*KO female (Figure 1E-G) and male rats (Figure 1H-J) compared to their controls (in both 1-hour and 4-hour average cases). Body weight was significantly increased in *Tgr5*KO female and *Tgr5*KO male rats compared to their respective controls (Figure S1A, S1H), although there was no difference in food and water intake between the groups (Figure S1B, S1C, Figure S1I, S1J). Kidney and liver weights, expressed as a percentage of body weight, were significantly reduced only in *Tgr5*KO female rats compared with their respective controls, whereas heart weight, expressed as a percentage of body weight, was significantly reduced only in *Tgr5*KO male rats compared with their controls (Figure S1D-S1F, Figure S1K-S1M). Spleen weight, expressed as a percentage of body weight, did not differ between groups in either sex (Figure S1G, S1N). Comprehensive laboratory animal monitoring system (CLAMS) determined a higher volume of oxygen consumption, increased volume of carbon dioxide production, and respiratory exchange ratio in both *Tgr5*KO female and *Tgr5*KO male rats compared to their respective controls (Figure S2A-S2F).

The serum metabolic and hepatic injury markers measured by the levels of serum triglyceride, total cholesterol, total bile acid, and alanine aminotransaminase (ALT) were not different between the groups in both sexes (Figure S3A-S3D, Figure S3G-S3J). However, serum alkaline phosphatase (ALP) was significantly lowered in *Tgr5*KO females compared to their controls, but no difference was observed in males (Figure S3E, S3K). Serum aspartate aminotransferase (AST) was significantly lowered in *Tgr5*KO males compared to their controls, but no difference was observed in females (Figure S3F, S3L). Twenty-four-hour urine volume and total protein excretion were comparable between the groups in both sexes (Figure S4A, S4B, Figure S4H, S4I). Cardiac function measured as ejection fraction, fractional shortening, cardiac output, stroke volume, and left ventricular mass via echocardiography revealed no significant differences between groups in either sex (Figure S4C, S4D, S4E, S4F, S4G, Figure S4J, S4K, S4L, S4M, S4N).

Wire myography in the dorsal aorta (DA) of females revealed no significant differences in the responses to any of the utilized vasoactive agents between the two groups (Figure S5A-S5F). However, in mesenteric resistance arteries (MRA), a trend for decreased Acetylcholine (ACh)-induced relaxation was observed in the *Tgr5*KO group, approaching statistical significance at higher concentrations (*p* = 0.06) (Figure S5K). No other differences were noted (Figure S5G-S5J, S5L). However, in the dorsal aortae of males, high potassium chloride (KCl, 120 mM) induced robust contraction in aortic rings, with a significantly greater response observed in the *Tgr5*KO group compared to controls (Figure S6A). When normalized to high-KCl contraction, phenylephrine (PE), and 5-hydroxytryptamine (5-HT) showed no differences in concentration-dependent contraction in aortic rings in both groups (Figure S6B, S6C). Pretreatment with Nω-Nitro-L-arginine methyl ester (L-NAME) significantly enhanced the maximal PE-induced constriction in the control group but not in *Tgr5*KO, suggesting impaired basal nitric oxide (NO) bioavailability and endothelial dysfunction in *Tgr5*KO vessels (Figure S6D). However, ACh- and sodium nitroprusside (SNP)-induced relaxation following PE precontraction was similar between groups, indicating intact endothelial-dependent and endothelial-independent relaxation in *Tgr5*KO vessels (Figure S6E, S6F). In the mesenteric resistance arteries of males, a trend for decreased ACh-induced relaxation in the *Tgr5*KO group was observed (Figure S6K). No other differences were noted (Figure S6G-S6J, S6L).

### Gut microbiota composition was remodeled in *Tgr5*KO rats

**Figure 2.**
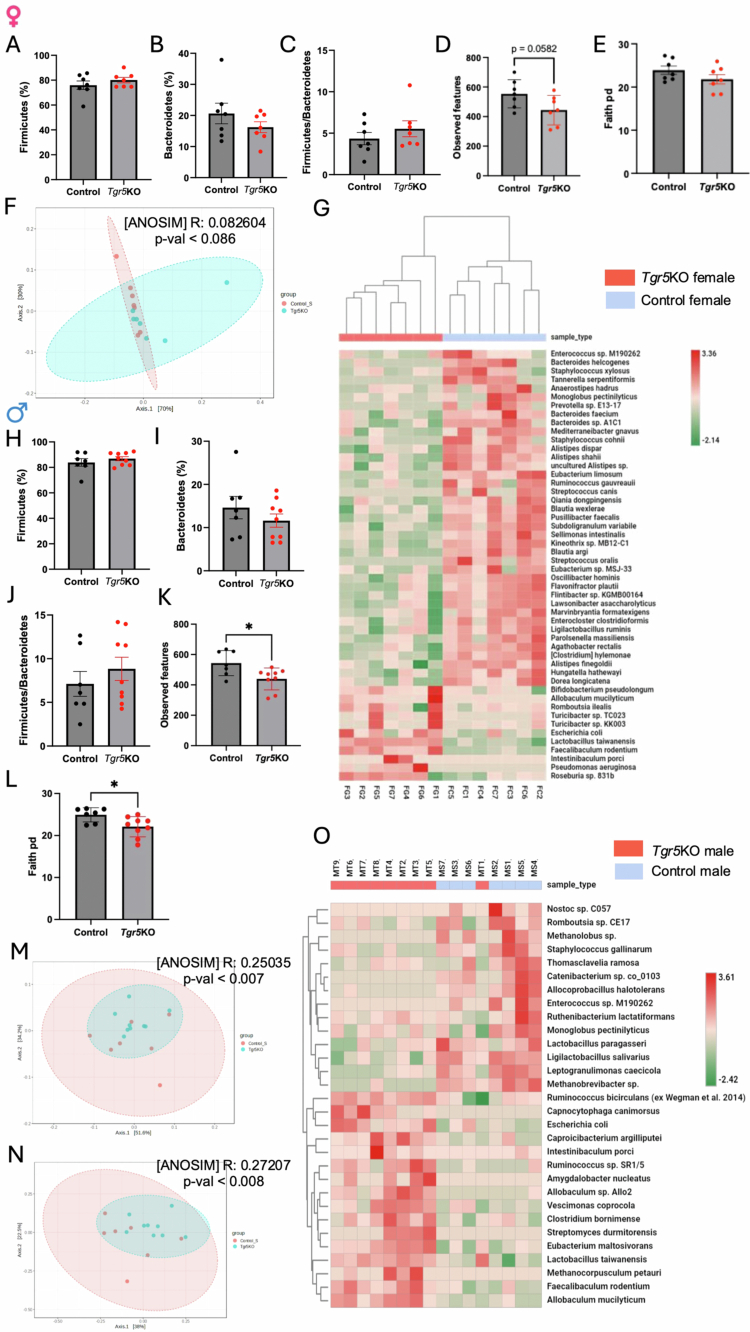
Gut microbiota composition was remodeled in *Tgr5*KO rats. In female rats: (A) Level of Firmicutes. (B) Level of Bacteroidetes. (C) Firmicutes/Bacteroidetes ratio. (D) Observed features. (E) Faith phylogenetic diversity. (F) Jensen–Shannon divergence. (G) Relative abundance of microbiota (Control: FC1-FC7, *Tgr5*KO: FG1-FG7). In male rats: (H) Level of Firmicutes. (I) Level of Bacteroidetes. (J) Firmicutes/Bacteroidetes ratio. (K) Observed features. (L) Faith phylogenic diversity. (M) Jensen–Shannon divergence. (N) Bray‒Curtis diversity. (O) Relative abundance of microbiota (Control: MS1-MS7, *Tgr5*KO: MT1-MT9). *Tgr5*KO: Takeda G-protein coupled receptor 5 knock out, *p* = *p*-value. *n* = 6-7 rats/group/sex. Unpaired, parametric two-tailed Student's *t* test with a 95% confidence interval was used to compare data between groups. Jensen–Shannon and Bray–Curtis *β*-diversity indexes were analyzed using ANOSIM. Cut-off of FDR < 0.05 was used to generate heat maps of relatively abundant microbiota. All data are mean ± SEM, *p < 0.05.

While 16S rRNA gene sequencing revealed no difference in the level of Firmicutes, Bacteroidetes, or Firmicutes/Bacteroidetes ratio in either female or male rats (Figure 2A-C and H-J), the *α*-diversity matrix observed features were lowered in *Tgr5*KO female and male rats compared to their controls (Figure 2D and K). Faith phylogenetic diversity was significantly reduced in *Tgr5*KO male rats but not in *Tgr5*KO females compared to their controls (Figure 2E and L). A significant shift in *β*-diversity, indexed by the Jensen–Shannon divergence, was observed between female *Tgr5*KO rats and their respective controls (Figure 2F). In male rats, *β*-diversity differences were evident by two other measures, the Jensen–Shannon divergence and Bray–Curtis metrices (Figure 2M and N). Whole genome sequencing revealed distinct microbiota composition between the two groups in both sexes. Among others, *Bifidobacterium pseudolongum, Allobaculum mucilyticulum, Romboutsia ilealis,* *Faecalibacterium rodentium,* and *Intestinibaculum porci,* were highly abundant in *Tgr5*KO females compared to the controls (Figure 2G). In male rats, among others, *Methanocorpusculum petauri*, *Faecalibaculum rodentium*, and *Allobaculum mucilyticum* were relatively abundant in *Tgr5*KO males compared to their controls (Figure 2O). Comparing female and male rats, we found *Faecalibaculum rodentium, Allobaculum mucilyticum, Intestinibaculum porci, Escherichia coli,* and *Lactobacillus taiwanensis* as common microbiota highly abundant in the *Tgr5*KO group compared to their respective controls. Furthermore, microbial diversity was more evident in *Tgr5*KO males compared to the female *Tgr5*KO group (Figure 2G and O).

### Secondary bile acid was significantly increased in *Tgr5*KO rats

**Figure 3.**
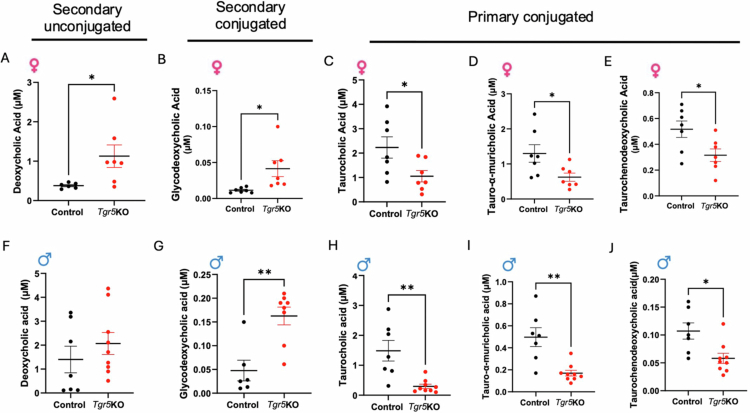
Secondary bile acid was significantly increased in *Tgr5*KO rats. In females (A) Deoxycholic acid. (B) Glycodeoxycholic acid. (C) Taurocholic Acid. (D) Tauro-*α*-muricholic acid. (E) Taurochenodeoxycholic acid. In males (F) Deoxycholic acid. (G) Glycodeoxycholic acid. (H) Taurocholic acid. (I) Tauro-*α*-muricholic acid. (J) Taurochenodeoxycholic acid. *Tgr5*KO: Takeda G-protein coupled receptor 5 knock out, GDCA: Glycodeoxycholic acid. *n* = 7–8 rats/group/sex. Unpaired, parametric two-tailed Student's *t* test with a 95% confidence interval was used to compare data between groups. All data are mean ± SEM, *p < 0.05, **p < 0.01.

The secondary unconjugated bile acid, deoxycholic acid, was significantly increased exclusively in *Tgr5*KO females but not in males (Figure 3A and F), whereas a secondary conjugated bile acid, glycodeoxycholic acid, was significantly increased in both female and male *Tgr5*KO rats compared to their respective controls (Figure 3B and G). Primary conjugated bile acids, taurocholic acid, tauro-*α*-muricholic acid, and taurochenodeoxycholic acid were significantly lowered in both female and male *Tgr5*KO rats compared to their respective controls (Figure 3C-E and Figure 3H-J). We previously reported that taurocholic acid is an anti-hypertensive metabolite.^4^ Despite a significant reduction of taurocholic acid in *Tgr5*KO groups, we observed the lowering of blood pressure, which raised our interest in the secondary conjugated bile acid, glycodeoxycholic acid.

### Supplementation with glycodeoxycholic acid significantly lowered blood pressure

**Figure 4.**
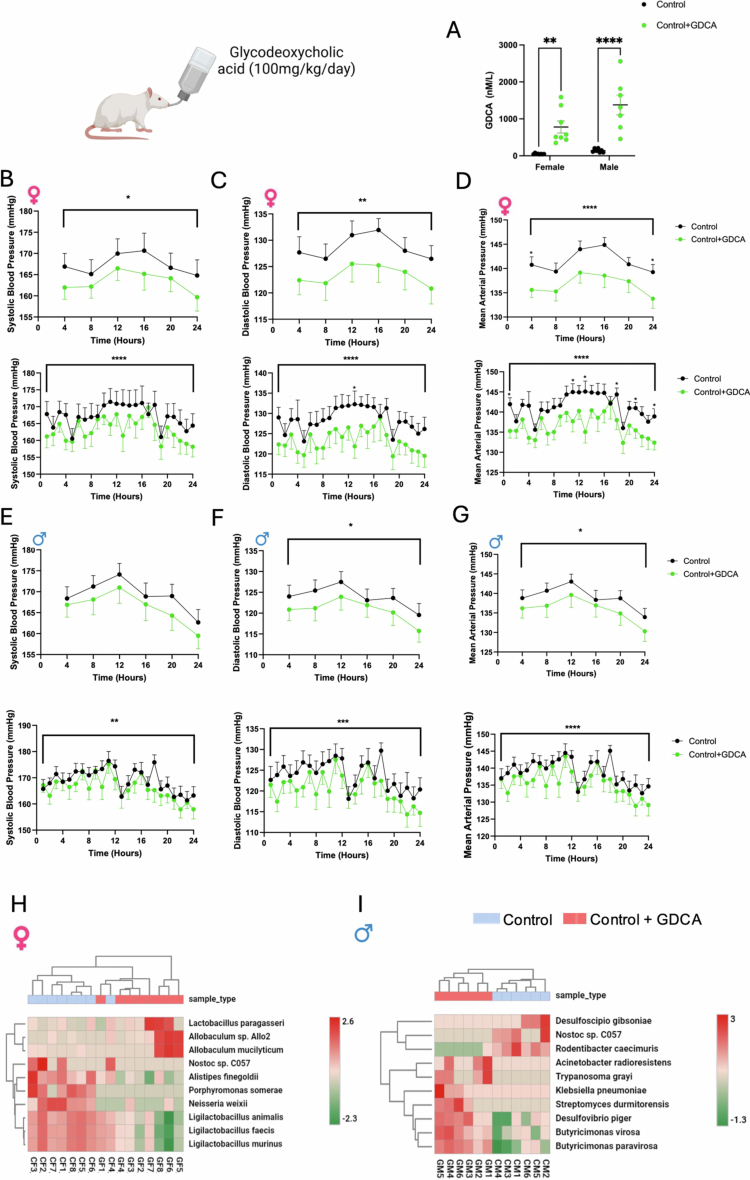
Glycodeoxycholic acid supplementation attenuated blood pressure and remodeled gut microbiota in control rats. (A) Serum concentration of glycodeoxycholic acid. 24-hour blood pressure reading in females (B) Systolic (Top panel: 4-hour averages, Bottom panel: 1-hour averages). (C), Diastolic (Top panel: 4-hour averages, Bottom panel: 1-hour averages). (D) Mean arterial pressure (Top panel: 4-hour averages, Bottom panel: 1-hour averages). 24-hour blood pressure reading in males (E) Systolic (Top panel: 4-hour averages, Bottom panel: 1-hour averages). (F) Diastolic (Top panel: 4-hour averages, Bottom panel: 1-hour averages). (G) Mean arterial pressure (Top panel: 4-hour averages, Bottom panel: 1-hour averages). (H) Relative abundance of microbiota in female rats (Control: CF1-CF8, Control + GDCA: GF1-GF8). (I) Relative abundance of microbiota in male rats (Control: CM1-CM6, Control + GDCA: GM1-GM6). *Tgr5*KO: Takeda G-protein coupled receptor 5 knock out, GDCA: Glycodeoxycholic acid. *n* = 6-8 rats/group/sex. Unpaired, parametric two-tailed Student's *t* test with a 95% confidence interval was used to compare data between groups (Figure 4A). For blood pressure measurements, repeated-measures 2-way ANOVA followed by Tukey multiple comparison test was used as the primary analysis to compare data between the groups at each time point. Ordinary 2-way ANOVA was used to assess the overall difference between the two groups. A cut-off of FDR < 0.05 was used to generate heat maps of relatively abundant microbiota. All data are mean ± SEM, *p < 0.05, **p < 0.01, ***p < 0.001, ****p < 0.0001.

Supplementation of glycodeoxycholic acid (100 mg/kg/d for four weeks) enhanced serum glycodeoxycholic acid level in both sexes (Figure 4A). Interestingly, systolic, diastolic, and mean arterial pressure were all significantly reduced in both sexes compared to their respective controls (Figure 4B-G). The glycodeoxycholic acid-supplemented group had no significant difference in body weight, food intake, and water intake (Figure S7A-S7C, S7H-S7J). No significant changes were observed in kidney, heart, liver, or spleen weights, expressed as a percentage of body weight, in either female or male rats compared to their respective controls (Figure S7D-S7G, S7K-S7N).

### Glycodeoxycholic acid supplementation remodeled gut microbiota and enhanced total bile acids

Whole genome sequencing revealed a distinct microbiota profile in glycodeoxycholic acid-supplemented female and male rats compared to their respective controls. *Allobaculum mucilyticum, Allobaculum sp*. *Allo 2*, and *Lactobacillus paragasseri* were found to be highly abundant in the glycodeoxycholic acid-supplemented females compared to the control group (Figure 4H). In males, among others, *Butyricimonas paravirosa, Butyricimonas virosa*, and *Streptomyces durmitorensis* were highly abundant in the glycodeoxycholic acid-supplemented group compared to the control group (Figure 4I). *Nostoc sp*. C057 was the only common bacterial species between the female and male control groups (Figure 4H and I).

Total bile acid and total cholesterol were significantly increased in the group supplemented with glycodeoxycholic acid in both sexes (Figure S8A, S8B, and Figure S8G, S8H), whereas triglyceride, ALT, AST, and ALP were comparable in both sexes between the groups (Figure S8C-S8F, and Figure S8I-S8L).

### Cecal microbiota transplantation lowered blood pressure in the control rats

**Figure 5.**
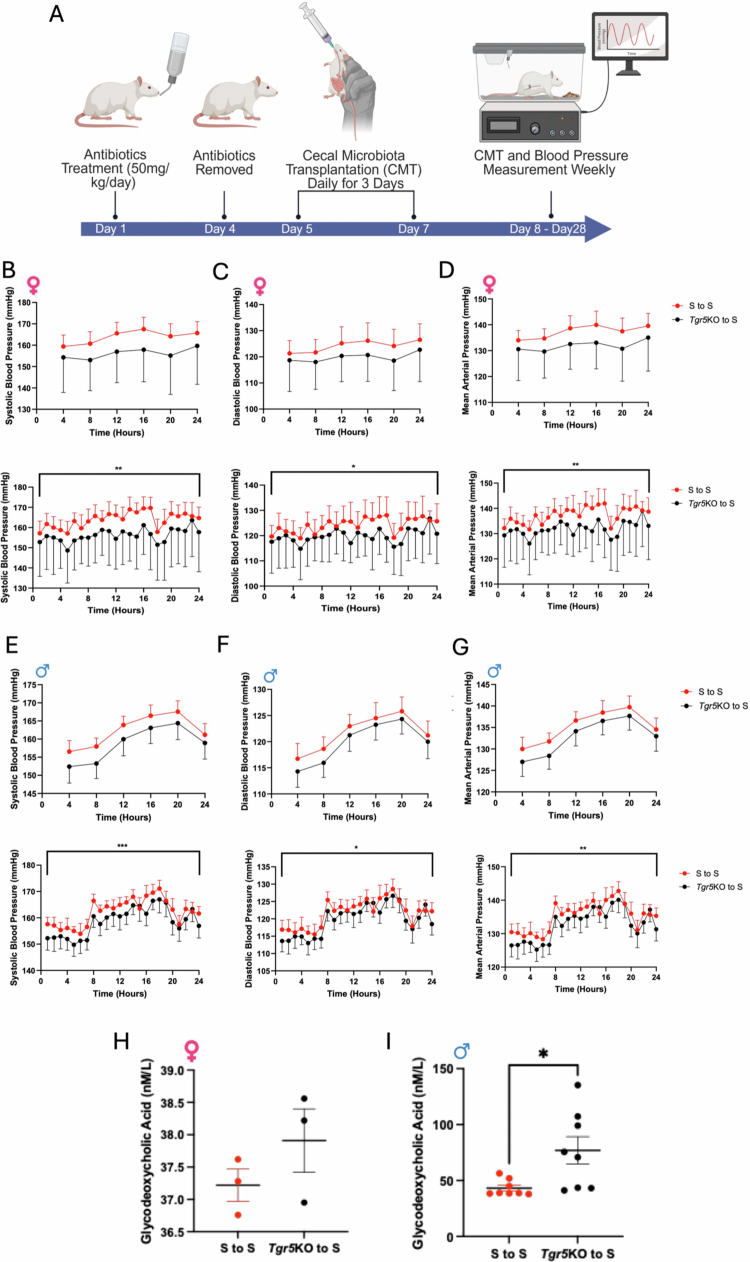
Cecal microbiota transplantation attenuated blood pressure in control rats. (A) Cecal microbiota transplantation workflow. 24-hour blood pressure reading in female rats (B) Systolic (Top panel: 4-hour averages, Bottom panel: 1-hour averages). (C) Diastolic (Top panel: 4-hour averages, Bottom panel: 1-hour averages). (D) Mean arterial pressure (Top panel: 4-hour averages, Bottom panel: 1-hour averages). 24-hour blood pressure reading in male rats (E) Systolic (Top panel: 4-hour averages, Bottom panel: 1-hour averages). (F) Diastolic (Top panel: 4-hour averages, Bottom panel: 1-hour averages). (G) Mean arterial pressure (Top panel: 4-hour averages, Bottom panel: 1-hour averages). Serum glycodeoxycholic acid concentration in female rats (H) and male rats (I). *Tgr5*KO: Takeda G-protein coupled receptor 5 knock out, S: Dahl Salt-sensitive rats (control). *n* = 3-7 rats/group/sex. For blood pressure measurements, repeated-measures 2-way ANOVA followed by Tukey multiple comparison test was used as the primary analysis to compare data between the groups at each time point. Ordinary 2-way ANOVA was used to assess overall difference between the two groups. Unpaired, parametric two-tailed Student’s *t* test with a 95% confidence interval was used to compare data between groups (Figure 5H and 5I). All data are mean ± SEM. *p < 0.05, **p < 0.01, ***p < 0.001.

Both female and male rats receiving cecal microbiota transplantation from donor *Tgr5*KO groups (Figure 5A) had significantly lower 1-hour average blood pressure compared to rats receiving cecal microbiota transplant from S rats (Figure 5B-G; bottom panels). However, 4-hour average blood pressure readings were not significantly different between the groups in both sexes (Figure 5B-G; top panels). Bile acid quantification revealed a significant increase in the glycodeoxycholic acid concentration in the male S rats receiving cecal microbiota from *Tgr5*KO rats compared to the control group (Figure 5I). In females, there was an increasing trend in the level of glycodeoxycholic acid in S rats receiving cecal microbiota transplantation from *Tgr5*KO rats compared to the control group (Figure 5H).

## Discussion

Hypertension is one of the major risk factors for cardiovascular diseases, accounting for millions of deaths worldwide. Therefore, there is an urgent need to develop novel therapeutic targets to combat hypertension. Published studies have reported a novel strategy of targeting the gut‒liver axis to attenuate hypertension.^4^ ^,^ ^5^ ^,^ ^42^ ^,^ ^43^ We previously discovered that taurocholic acid, a primary conjugated bile acid, is an antihypertensive metabolite.^4^ Bile acids are the major metabolites transacted between the host and the microbiota and are increasingly recognized as signaling molecules that influence a wide range of cardiovascular and metabolic processes.^44-47^

Among the receptors of these bile acids, TGR5 is a major secondary bile acid receptor and has been investigated for its role in energy homeostasis, glucose metabolism, and as an important modulator of immune and inflammatory response balance.^48^ ^,^ ^49^ Recent studies have begun to implicate TGR5 in blood pressure regulation. Activation of TGR5 by lithocholic acid was reported to attenuate blood pressure in DOCA-salt-induced hypertensive mice, whereas pharmacological TGR5 knockout in the same model increased blood pressure.^50^ Similarly, TGR5 activation by taurocholic acid was reported to attenuate blood pressure in spontaneously hypertensive mice.^51^ However, these prior studies relied on pharmacological TGR5 manipulation in murine models and did not examine the endogenous contribution of TGR5 to blood pressure regulation through gut microbiota remodeling in a genetically hypertensive rat model. Furthermore, whether the antihypertensive effects of the specific secondary bile acids extend beyond canonical TGR5 activation, and are therefore observed regardless of TGR5 signaling status, remained unresolved. The present study addresses these gaps through three independent and convergent experimental approaches: genetic TGR5 deletion in the background of the S rat by CRISPR/Cas9, direct glycodeoxycholic acid supplementation, and cecal microbiota transplantation in S rat to demonstrate that glycodeoxycholic acid is a novel gut‒liver axis-derived antihypertensive metabolite whose blood pressure-lowering effects are not exclusively contingent on TGR5 signaling status.

Twenty-four-hour blood pressure readings demonstrated significant lowering of systolic, diastolic, and mean arterial pressure in *Tgr5*KO rats in both sexes. *Tgr5*KO female and male rats displayed increased body weight and carbohydrate utilization, indicated by a higher respiratory exchange ratio (RER), as the primary energy substrate compared to their respective controls. Previously published studies reported that TGR5, upon getting signals from bile acids, regulates glucose and energy metabolism and promotes energy consumption of brown adipose tissue to achieve weight loss.^16^ ^,^ ^17^ ^,^ ^19^ However, the absence of TGR5-signaling has been reported to increase body weight in *Tgr5*KO mice,^52^ which was consistent with the findings of our study.

Blood pressure is regulated by multiple tissues, including the liver, heart, kidney, and vasculature.^53-60^ In the current study, we examined renal, hepatic, cardiac, and vascular function between the control and *Tgr5*KO groups in both sexes. While we observed a significant reduction in kidney and liver weight to body weight ratios in *Tgr5*KO females and heart weight to body weight ratio in *Tgr5*KO males, renal, hepatic, and cardiac functional parameters did not differ between groups. The preserved cardiac structure and pumping capacity despite the reduced heart weight to body weight ratio in *Tgr5*KO males suggest that cardiac function is not the primary driver of the reduction in blood pressure. Overall, these findings indicate that alterations in renal, cardiac, and hepatic function are unlikely to be the primary drivers of the reduced blood pressure observed in the *Tgr5*KO rats.

Interestingly, ALP and AST levels were significantly reduced in *Tgr5*KO females and *Tgr5*KO males, respectively, suggesting a potential reduction in hepatic stress, although further studies are needed to confirm the functional significance. However, previous studies in mice have reported that the absence of TGR5 can lead to cholestasis, liver injury, and inflammation.^61-63^

Vascular dysfunction shifts the vascular system toward higher tone, stiffness, and inflammation, all of which contribute to elevated blood pressure and the development of hypertension.^64^ Previous study has shown that activation of TGR5 promotes endothelial relaxation and protects against vascular injury.^65^ Consistently, in this study, we observed features of endothelial dysfunction in *Tgr5*KO rats. The vascular NO deficit in *Tgr5*KO rats was anatomically restricted and did not produce a generalized vascular dysfunction, as evidenced by preserved endothelial-dependent and endothelial-independent relaxation. The primary antihypertensive mechanism instead operates through gut microbiota remodeling and glycodeoxycholic acid-mediated systemic signaling, as independently confirmed by glycodeoxycholic acid supplementation and cecal microbiota transplantation experiments.

Previous studies have reported alterations in gut microbiota composition in *Tgr5*KO mice.^11^ ^,^ ^24^ In this study, we observed a modest but statistically significant shift in the gut microbial composition as evidenced by differences in *α*-diversity, *β*-diversity, and relative microbial abundance between the groups in both sexes; however, effect sizes were modest, and there was substantial overlap in community composition between the groups, suggesting limited ecological separation. The Firmicutes/Bacteroidetes ratio was included and evaluated as a descriptive summary only and not interpreted as an indicator of microbiome health. As TGR5 is a secondary bile acid receptor and secondary bile acids are produced by gut microbiota, the shift in gut microbiota composition suggested the possible alteration in the bile acid composition between the groups.

Quantitative bile acid profiling showed a marked increase in the secondary conjugated bile acid glycodeoxycholic acid and a reduction in taurine-conjugated primary bile acids in both female and male *Tgr5*KO rats. The impact of TGR5 deletion on total bile acid pool and composition remains inconclusive, as published studies have reported variable outcomes.^52^ ^,^ ^66-68^

Given these findings in the *Tgr5*KO rat model, it is important to consider species-specific differences in bile acid metabolism. Notably, the predominant mode of bile acid conjugation differs between species: glycine conjugation predominates in humans, whereas taurine conjugation is the primary form in rodents.^4^ ^,^ ^69^ This difference between species suggests that increasing glycine-conjugated bile acids, such as glycodeoxycholic acid, could be explored as a novel strategy to attenuate hypertension in humans, potentially through bile-acid-mediated effects on metabolic or vascular functions. The consistent and sex-independent elevation of glycodeoxycholic acid in *Tgr5*KO rats, combined with its biological relevance to human physiology as a glycine-conjugated bile acid, provided the rationale for directly testing its antihypertensive potential through supplementation in control S rats. Glycodeoxycholic acid supplementation significantly enhanced systemic glycodeoxycholic acid levels and lowered blood pressure in both sexes, supporting its potential translational relevance to humans. However, data from the *Tgr5*KO rats, wherein a lowering of blood pressure was observed despite the loss of TGR5 signaling, serve as evidence for glycodeoxycholic acid to lower blood pressure independent of TGR5 signaling. Nonetheless, it remains possible that other bile acids may also influence the blood pressure through TGR5-independent or compensatory pathways. Future studies examining the role of other specific bile acids could provide additional insight into bile acid mechanisms in the modulation of blood pressure in *Tgr5*KO and control rats.

Serum biochemical analyzes determined a significant increase in the level of cholesterol and total bile acid pool without any effect on the liver injury markers in both female and male rats supplemented with glycodeoxycholic acid compared to their respective controls. As secondary bile acids are formed from primary bile acids, an enhanced availability of secondary bile acids may have negatively regulated the production of primary bile acids via inhibition of cholesterol breakdown.

Published reports have documented the relationship between bile acids and gut microbiota composition.^7^ ^,^ ^70^ ^,^ ^71^ Metagenomic sequencing revealed distinct microbiota composition in both glycodeoxycholic acid-supplemented female and male rats compared to their respective controls. *Allobaculum mucilyticum* was identified as the only common taxon shared among *Tgr5*KO female and *Tgr5*KO male rats and glycodeoxycholic acid-supplemented female rats. While a shift in the genus *Allobaculum* has been previously reported to influence blood pressure,^72^ ^,^ ^73^ the role of *Allobaculum mucilyticum* has not yet been directly examined in the context of blood pressure regulation. Future targeted investigations are needed to find a potential link between *Allobaculum mucilyticum*, glycodeoxycholic acid, and blood pressure. *Nostoc sp. C057* was identified as the sole common taxon shared between control males in the *Tgr5*KO study and both control female and male rats in the glycodeoxycholic acid-supplemented study.

To further strengthen the role of gut microbiota in blood pressure regulation, we performed cecal microbiota transplantation following our previously published protocol.^29^ We treated recipient rats in both groups with antibiotics to deplete the resident microbiota before the cecal microbiota transplantation. Previously published studies have reported the influence on blood pressure in the recipient via cecal or fecal microbiota transplantation.^29^ ^,^ ^74-76^ Consistent with the published studies, microbiota transplant from donor *Tgr5*KO rats to control S rats significantly lowered blood pressure in the recipient rats in both sexes. While 4-hour average blood pressure data analysis did not reveal a statistically significant difference between groups, using 1-hour average data points revealed a significant difference between the groups. The lack of statistical significance in the 4-hour interval analysis, compared to the 1-hour analysis, is likely due to temporal aggregation of the data. Averaging across broader time windows reduces the number of data points and can obscure underlying variability, thereby diminishing statistical power and masking differences detectable at finer temporal resolution. The observed blood pressure differences implied that the remodeled gut microbiota as a result of knocking out TGR5 is the primary driver of blood pressure regulation. Moreover, the key finding that glycodeoxycholic acid is elevated in the control S rats by the transplantation of the remodeled gut microbiota from *Tgr5*KO rats is strong evidence to prioritize glycodeoxycholic acid as a key mediator of the gut microbiota-driven blood pressure-lowering effect.

Although the findings of our study are intriguing, there are some limitations. The rat strains were bred separately, and therefore, the baseline microbiome differences related to housing and environmental exposure cannot be fully excluded. The animals were, however, maintained in the same facility under identical housing, diet, and handling conditions during the entire duration of the study to mitigate the possible differences in the microbial composition. The lack of littermate controls or cage-swapping strategies to minimize these potential differences is a limitation of the study and may help explain the modest differences observed in microbiota composition between groups. The observation that blood pressure is reduced in both *Tgr5*KO rats and in glycodeoxycholic acid-supplemented S rats with intact TGR5 suggests that glycodeoxycholic acid may exert its effects through additional compensatory pathways beyond TGR5 signaling. It remains plausible that glycodeoxycholic acid may act through additional mechanisms, possibly involving crosstalk with another major bile acid receptor, Farnesoid X Receptor (FXR) signaling, endothelial signaling, or alteration in gut microbial metabolites, which collectively influence blood pressure regulation.^5^ ^,^ ^77^ ^,^ ^78^ Given that TGR5 is expressed across intestinal epithelial cells, immune cells, and metabolic tissues, these pathways may also contribute to systemic blood pressure regulation. Also, future studies involving receptor-specific agonists or antagonists and mechanistic assays will be necessary to clarify the relative contributions of specific receptor-dependent and receptor-independent mechanisms underlying blood pressure regulation by glycodeoxycholic acid. Another limitation of our study is the small sample size of female rats in the cecal microbiota transplantation study. Although the blood pressure-lowering effect was observed in female rats receiving cecal microbiota transplantation from *Tgr5*KO rats despite no significant increase in the glycodeoxycholic acid level, future studies with a larger sample size may increase the statistical power to demonstrate whether the blood pressure-lowering effect is either glycodeoxycholic acid-mediated or not.

In conclusion, this study provides the first genetic loss-of-function evidence that TGR5 deletion in the S rat remodels the gut microbiota, elevates the secondary conjugated bile acid glycodeoxycholic acid, and lowers blood pressure. Importantly, the consistent blood pressure-lowering effect of glycodeoxycholic acid both in the absence and presence of TGR5 signaling suggests that its antihypertensive effects are not exclusively contingent on canonical TGR5 activation. By modulating microbiota, glycodeoxycholic acid exerts potent antihypertensive effects while preserving systemic metabolic homeostasis. Targeting the gut‒liver axis and exploiting bile acid metabolism, specifically via enhancing glycodeoxycholic acid as an endogenous antihypertensive metabolite, constitutes a new strategy to ameliorate hypertension.

## Supplementary Material

Fig S5.jpg Fig S5.jpg

Graphical abstract publication license BioRender.pdf Graphical abstract publication license BioRender.pdf

Fig S4.jpg Fig S4.jpg

Fig S8.jpg Fig S8.jpg

Fig S3.jpg Fig S3.jpg

Fig S1.jpg Fig S1.jpg

Fig S7.jpg Fig S7.jpg

Fig S6.jpg Fig S6.jpg

Fig S2.jpg Fig S2.jpg

Supplementary Table S1.docx

Supplementary Figure Legends Supplementary Figure Legends

## Data Availability

All data and materials have been included within the manuscript. 16S rRNA gene sequencing data have been deposited into the NCBI Sequence Read Archive (SRA) under BioProject PRJNA140577 and Nanopore whole-genome sequencing data have been deposited into the NCBI SRA under BioProject PRJNA1413405.

## Abbreviations

ACh: Acetylcholine
ALP: Alkaline phosphatase
ALT: Alanine aminotransaminase
AST: Aspartate aminotransferase
CLAMS: Comprehensive laboratory animal monitoring system
DOCA: Deoxycorticosterone acetate
DA: Dorsal aorta
DBP: Diastolic blood pressure
GDCA: Glycodeoxycholic acid
KCl: Potassium chloride
L-NAME: Nω-Nitro-L-arginine methyl ester
LVEDV: Left ventricular end-diastolic volume
LVESV: Left ventricular end-systolic volume
LVID, d: Left ventricular internal dimensions diastole
LVID, s: Left ventricular internal dimensions systole
LVI: Left ventricular mass index
MAP: Mean arterial pressure
MRA: Mesenteric resistance artery
PE: Phenylephrine
RER: Respiratory exchange ratio
SBP: Systolic blood pressure
SNP: Sodium nitroprusside
TBA: Total bile acid
TGR5: Takeda G-protein coupled receptor 5
*Tgr5*KO: Takeda G-protein coupled receptor 5 knock out
VCO_2_: Volume of carbon dioxide produced
VO_2_: Volume of oxygen consumed
5-HT: 5-hydroxytryptamine
