# Supplementary figures and images for: Gut microbiome drives glycodeoxycholic acid-mediated attenuation of hypertension
Source: Gut Microbes. 2026 Jun 24;18(1):2691346. doi: 10.1080/19490976.2026.2691346 (PMC13313272; doi:10.1080/19490976.2026.2691346)

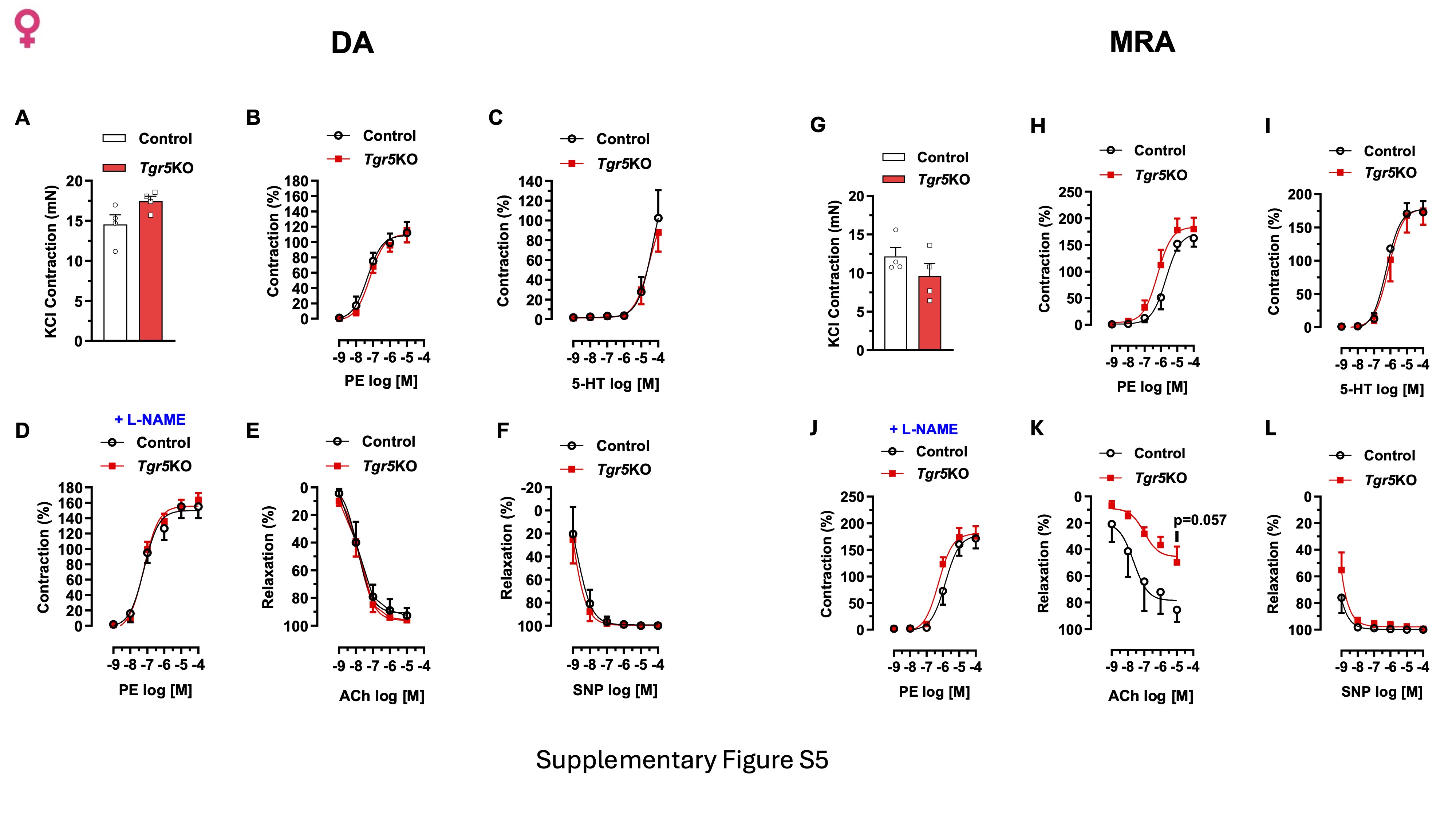

Supplement: Fig S5.jpg [file KGMI_A_2691346_SM5940.jpg]

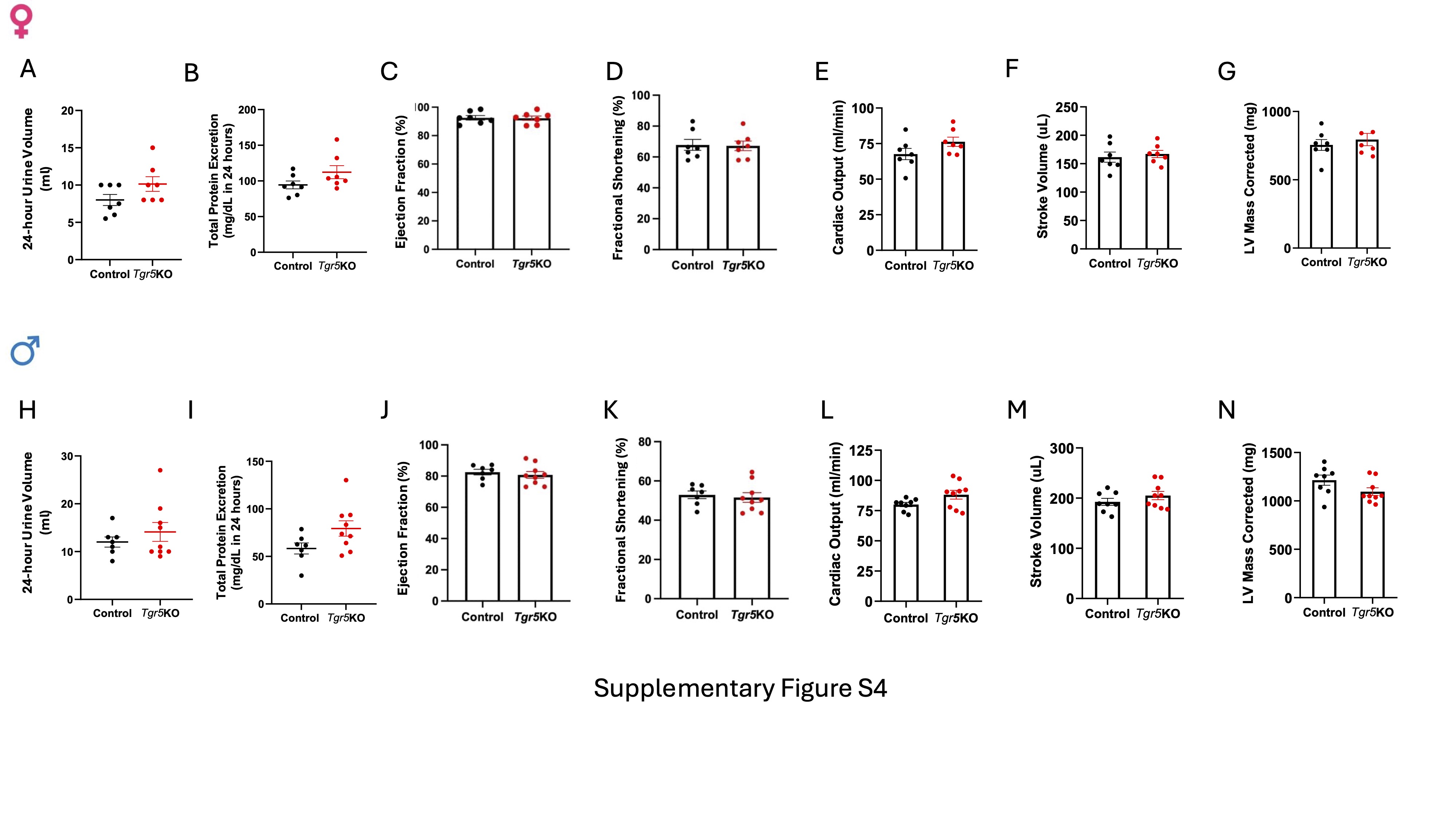

Supplement: Fig S4.jpg [file KGMI_A_2691346_SM5942.jpg]

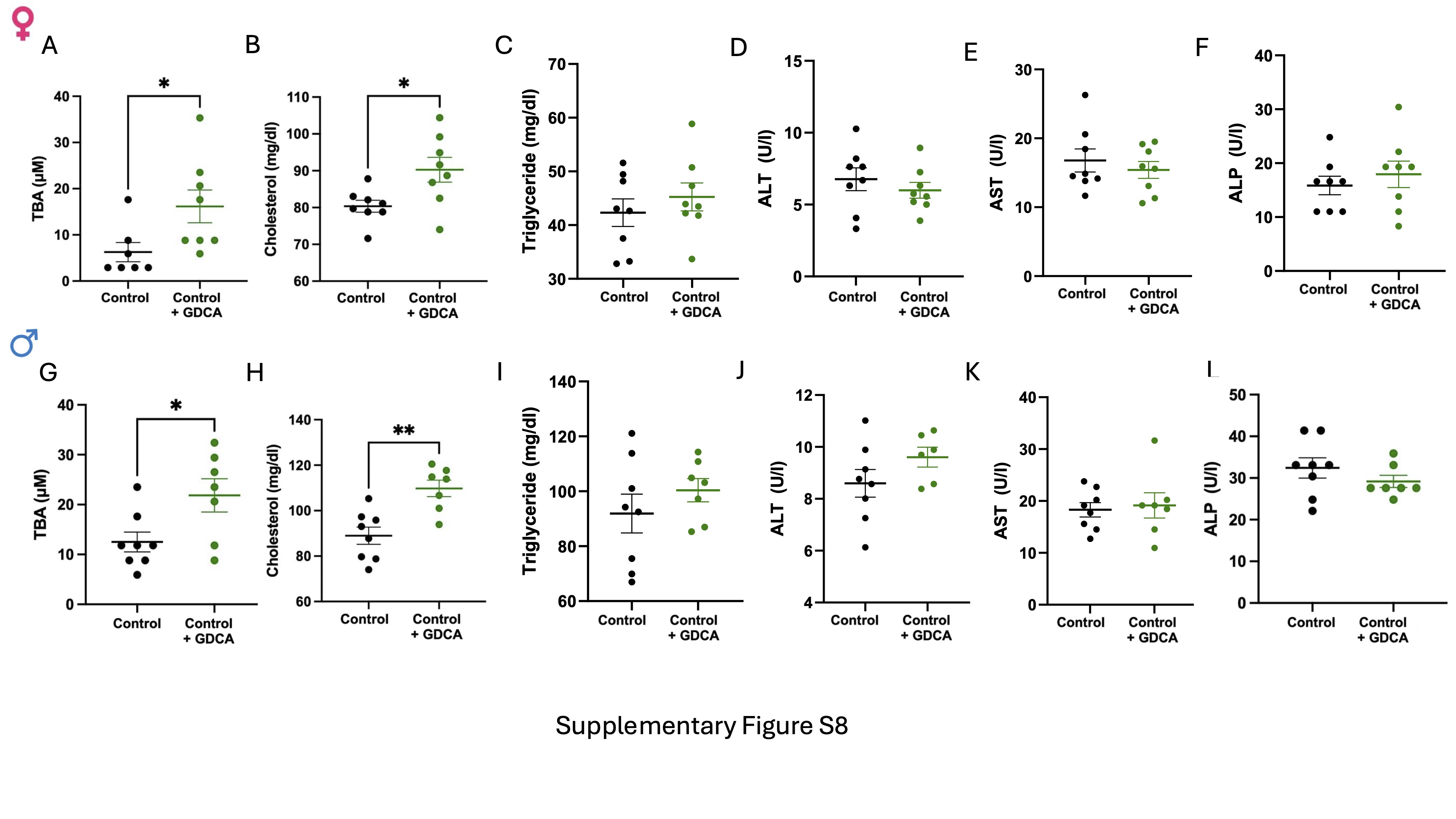

Supplement: Fig S8.jpg [file KGMI_A_2691346_SM5943.jpg]

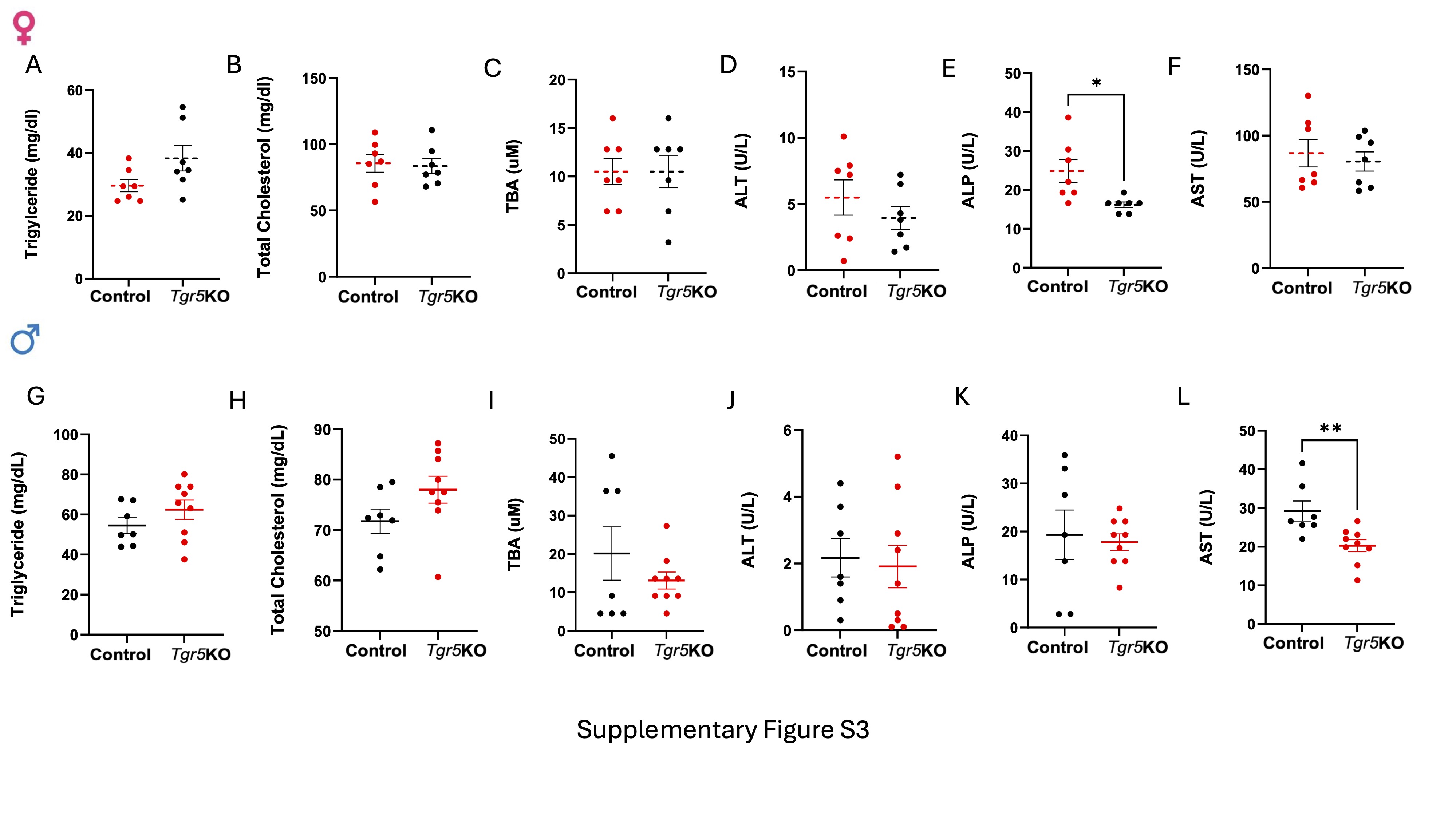

Supplement: Fig S3.jpg [file KGMI_A_2691346_SM5944.jpg]

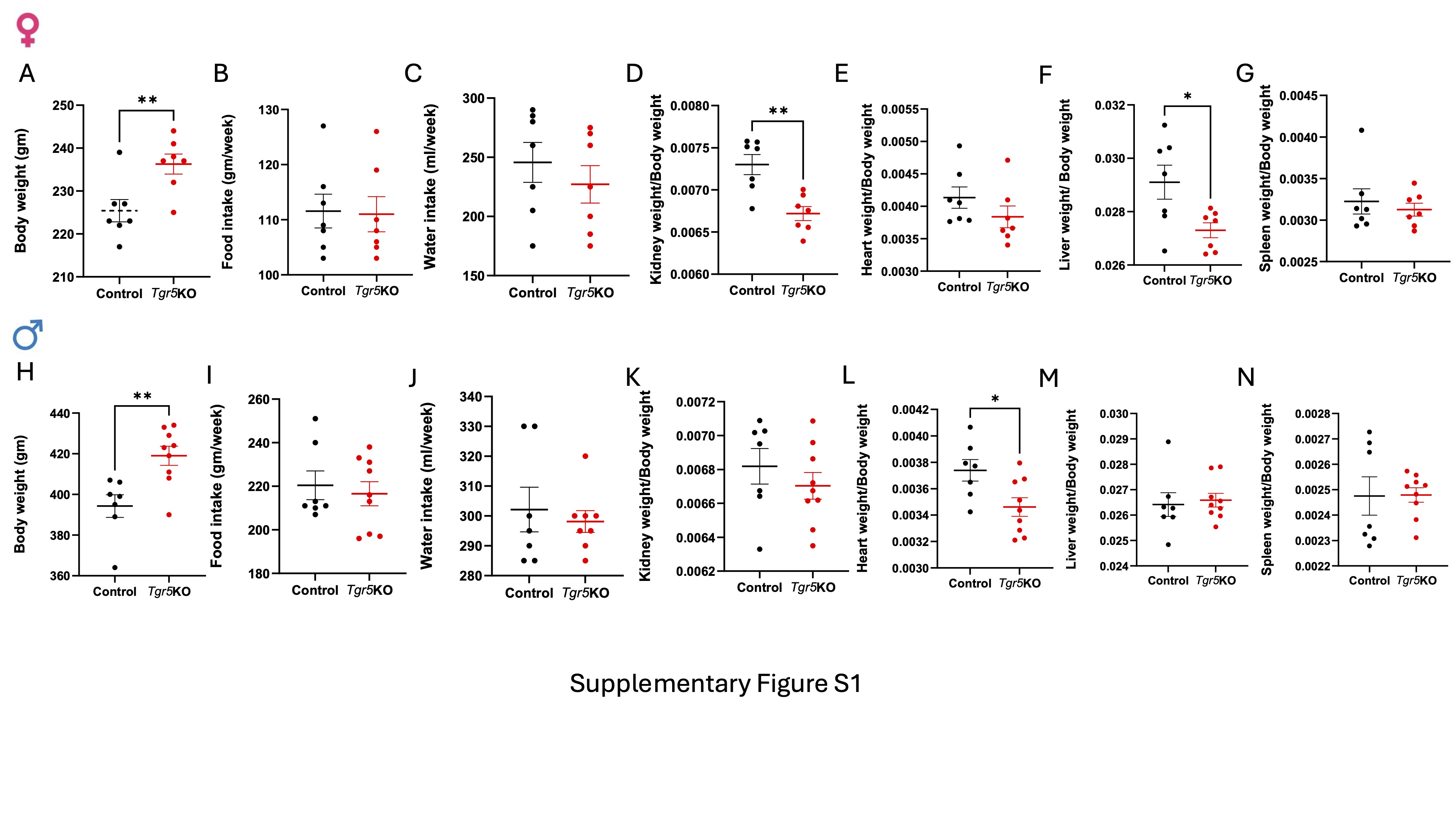

Supplement: Fig S1.jpg [file KGMI_A_2691346_SM5945.jpg]

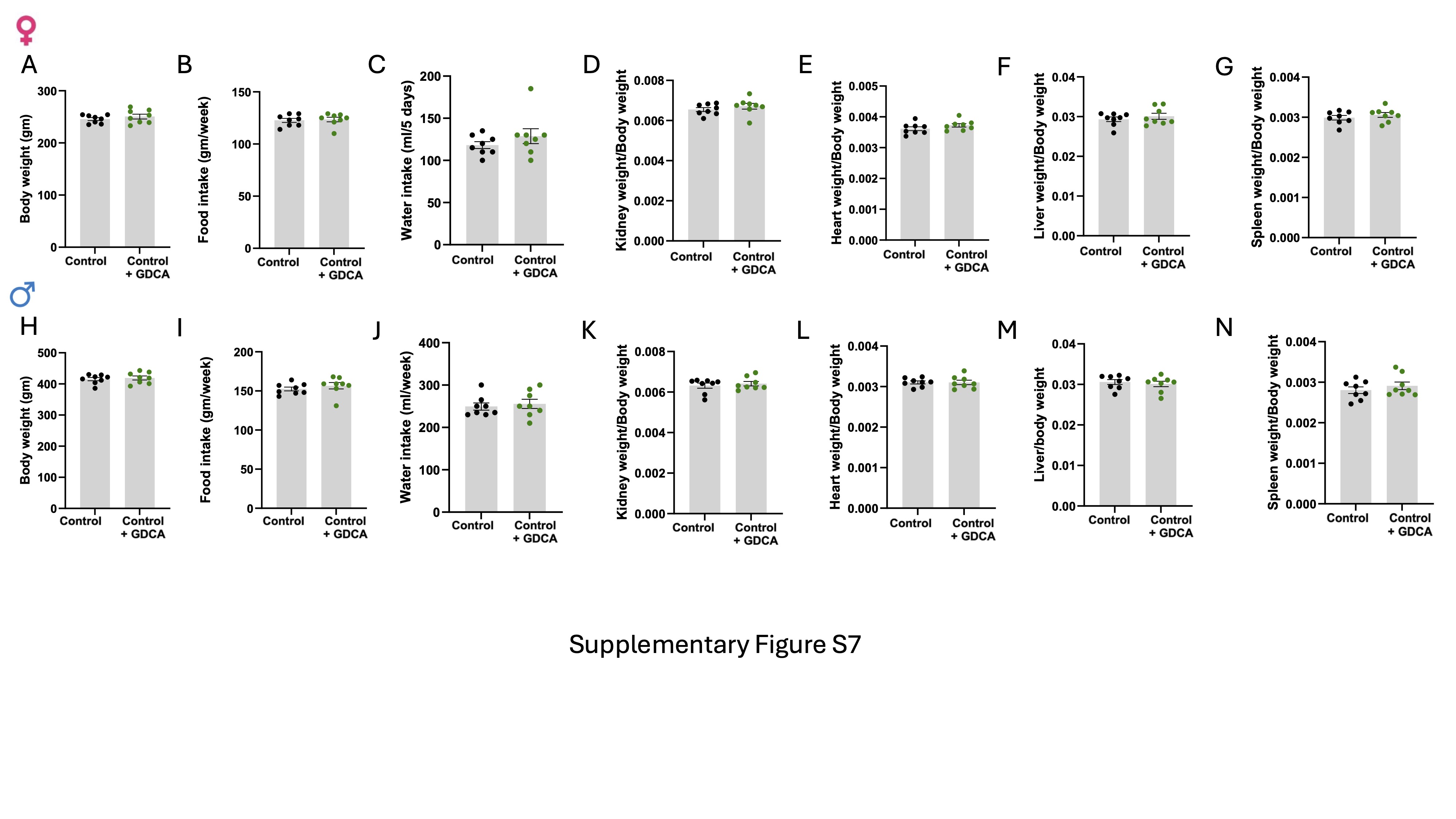

Supplement: Fig S7.jpg [file KGMI_A_2691346_SM5946.jpg]

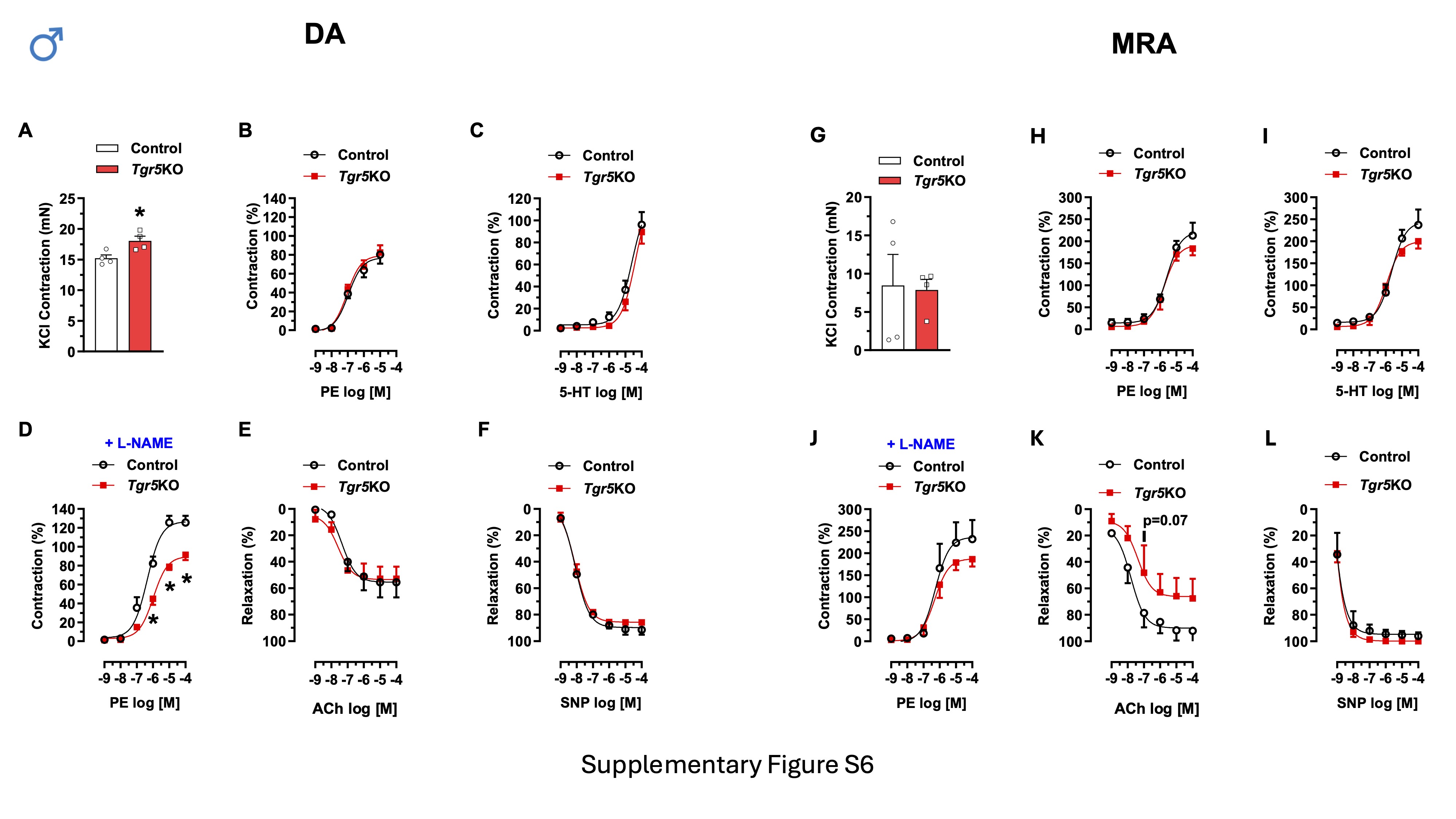

Supplement: Fig S6.jpg [file KGMI_A_2691346_SM5947.jpg]

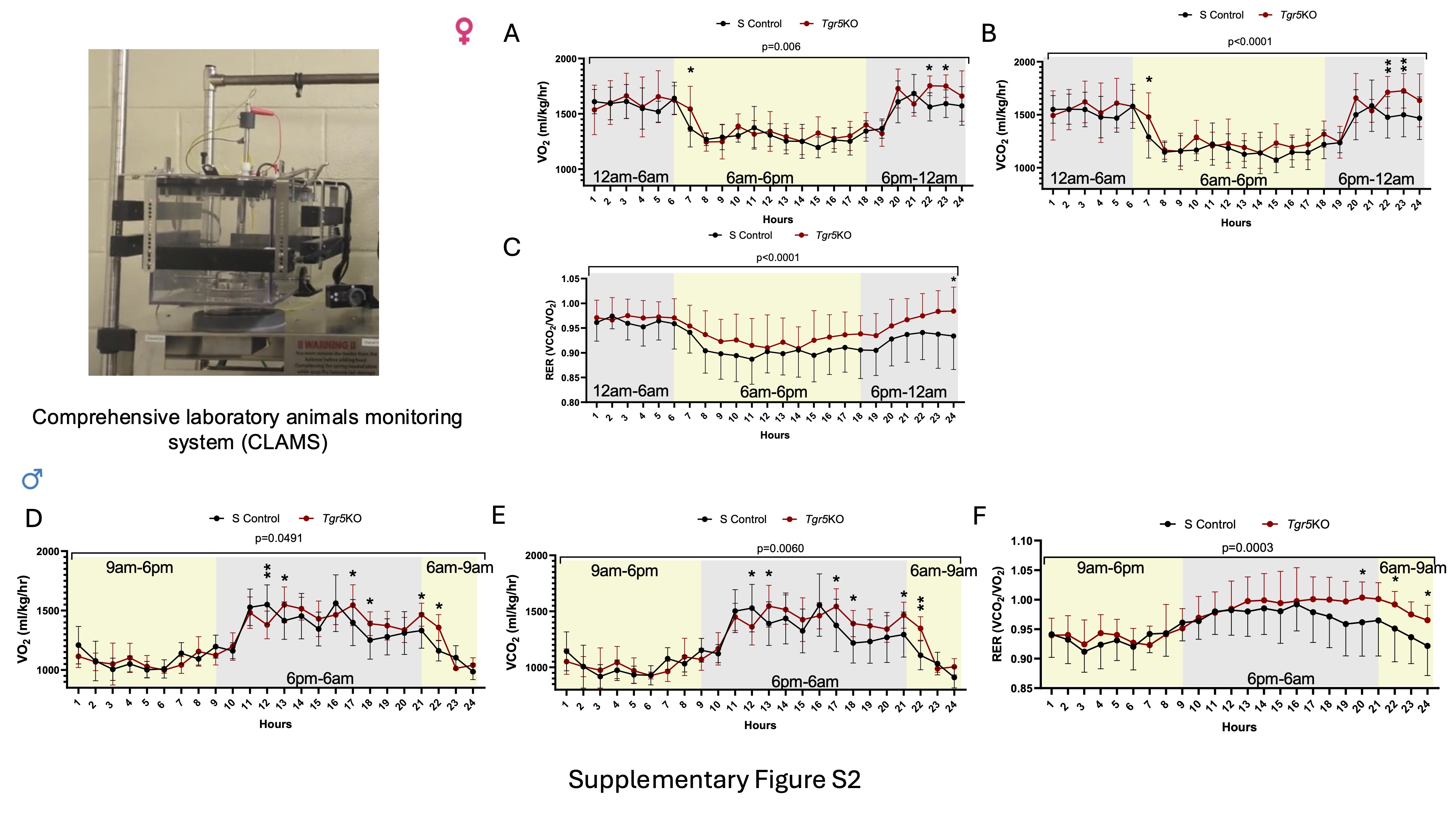

Supplement: Fig S2.jpg [file KGMI_A_2691346_SM5948.jpg]
