# Supplementary material for: Gut microbiome drives glycodeoxycholic acid-mediated attenuation of hypertension
Source: Gut Microbes. 2026 Jun 24;18(1):2691346. doi: 10.1080/19490976.2026.2691346 (PMC13313272; doi:10.1080/19490976.2026.2691346)
Supplement: Supplementary Table S1.docx [file KGMI_A_2691346_SM5949.docx]

**Supplementary Table**

**Table S1.** The forward and reverse sequences for the tested transcripts.

| **Gene** | **Forward sequence (5’-3’)** | **Reverse sequence (3’-5’)** |
| --- | --- | --- |
| *Tgr5* | CCACCACTAGGGCCTGTAAC | CCTCGAAGCACTTGTAGCCA |
| *β-Actin* | AGGGAAATCGTGCGTGACAT | TGGCCATCTCTTGCTCGAAG |
