# Supplementary material for: Gut microbiome drives glycodeoxycholic acid-mediated attenuation of hypertension
Source: Gut Microbes. 2026 Jun 24;18(1):2691346. doi: 10.1080/19490976.2026.2691346 (PMC13313272; doi:10.1080/19490976.2026.2691346)
Supplement: Supplementary Figure Legends [file KGMI_A_2691346_SM3697.docx]

**Supplementary Figure Legends**

**Figure S1:** Body weight was significantly increased in *Tgr5*KO rats. In females (A) Body weight. (B) Food-intake. (C) Water intake. (D) Kidney weight/body weight ratio. (E) Heart weight/body weight ratio. (F) Liver weight/body weight ratio. (G) Spleen weight/body weight ratio. In males (H) Body weight. (I) Food-intake. (J) Water intake. (K) Kidney weight/body weight ratio. (L) Heart weight/body weight ratio. (M) Liver weight/body weight ratio. (N) Spleen weight/body weight ratio. *Tgr5*KO: Takeda G-protein coupled receptor 5 knock out. n = 7-9 rats/group/sex. Unpaired, parametric two-tailed Student’s *t* test with a 95% confidence interval was used to compare data between groups. All data are mean ± SEM, *p<0.05, **p<0.01.

**Figure S2:** *Tgr5*KO rats showed enhanced respiratory exchange ratio. In females (A) Volume of oxygen consumption. (B) Volume of carbon dioxide production. (C) Respiratory exchange ratio. In males (D) Volume of oxygen consumption. (E) Volume of carbon dioxide production. (F) Respiratory exchange ratio. *Tgr5*KO: Takeda G-protein coupled receptor 5 knock out, VO_2_: Volume of oxygen consumed, VCO_2_: Volume of carbon dioxide produced, RER: Respiratory exchange ratio. n = 6-7 rats/group/sex. p = p-value. Data was analyzed with 2-way ANOVA with Fisher’s least significant difference test. All data are mean ± SEM, *p<0.05, **p<0.01.

**Figure S3:** Hepatic function was comparable in *Tgr5*KO rats. In females (A) Serum triglyceride level. (B) Serum total cholesterol level. (C) Serum TBA level. (D) Serum ALT level. (E) Serum ALP level. (F) Serum AST level. In males (G) Serum triglyceride level. (H) Serum total cholesterol level. (I) Serum TBA level. (J) Serum ALT level. (K) Serum ALP level. (L) Serum AST level. *Tgr5*KO: Takeda G-protein coupled receptor 5 knock out, TBA: Total bile acid, ALT: Alanine aminotransaminase, ALP: Alkaline phosphatase, AST: Aspartate aminotransferase. n = 7-9 rats/group/sex. Unpaired, parametric two-tailed Student’s *t* test with a 95% confidence interval was used to compare data between groups. All data are mean ± SEM, *p<0.05, **p<0.01.

**Figure S4:** TGR5 deficiency does not affect renal function and cardiac function in rats. In females (A) 24-hour urine volume. (B) Total urinary protein excretion. (C) Ejection fraction. (D) Fractional shortening. (E) Cardiac output. (F) Stroke volume. (G) Left ventricular mass. In males (H) 24-hour urine volume. (I) Total urinary protein excretion. (J) Ejection fraction. (K) Fractional shortening. (L) Cardiac output. (M) Stroke volume. (N) Left ventricular mass. *Tgr5*KO: Takeda G-protein coupled receptor 5 knock out. LV: Left ventricle. n = 7-9 rats/group/sex. Unpaired, parametric two-tailed Student’s *t* test with a 95% confidence interval was used to compare data between groups. All data are mean ± SEM.

**Figure S5:** *Tgr5*KO female rats exhibit endothelial dysfunction. Vascular reactivity analysis of females utilizing dorsal aortic rings (A-F) and mesenteric arterial rings (G-L). (A, G) Contraction induced by high potassium chloride solution (KCl, 120mM). (B, H) Contractions induced by phenylephrine (PE, 10^-9^-10^-4^ mol/L [M]). (C, I) Contractions induced by 5-hydroxytryptamine (5-HT, 10^-9^-10^-4^ M). (D, J) Effects of pretreatment with Nω-Nitro-L-arginine methyl ester (L-NAME, 100μM, 30 min) on phenylephrine-induced contractions. (E, K) Relaxations induced by acetylcholine (ACh, 10^-9^-10^-4^ M) after precontraction with phenylephrine (10^−7^ M). (F, L) Relaxations induced by sodium nitroprusside (SNP, 10^-9^-10^-4^ M) after precontraction with phenylephrine (10^−7^ M). *Tgr5*KO: Takeda G-protein coupled receptor 5 knock out, DA: Dorsal aorta, MRA: Mesenteric resistance artery. n = 4 rats/group, p = p-value. Data were analyzed with 2-way ANOVA with Fisher’s least significant difference test. All data are mean ± SEM.

**Figure S6:** *Tgr5*KO male rats exhibit endothelial dysfunction. Vascular reactivity analysis of males utilizing dorsal aortic rings (A-F) and mesenteric arterial rings (G-L). (A, G) Contraction induced by high potassium chloride solution (KCl, 120mM). (B, H) Contractions induced by phenylephrine (PE, 10^-9^-10^-4^ mol/L [M]). (C, I) Contractions induced by 5-hydroxytryptamine (5-HT, 10^-9^-10^-4^ M). (D, J) Effects of pretreatment with Nω-Nitro-L-arginine methyl ester (L-NAME, 100μM, 30 min) on phenylephrine-induced contractions. (E, K) Relaxations induced by acetylcholine (ACh, 10^-9^-10^-4^ M) after precontraction with phenylephrine (10^−7^ M). (F, L) Relaxations induced by sodium nitroprusside (SNP, 10^-9^-10^-4^ M) after precontraction with phenylephrine (10^−7^ M). *Tgr5*KO: Takeda G-protein coupled receptor 5 knock out, DA: Dorsal aorta, MRA: Mesenteric resistance artery. n = 4 rats/group, p = p-value. Data were analyzed with 2-way ANOVA with Fisher’s least significant difference test. All data are mean ± SEM. *p<0.05.

**Figure S7:** Glycodeoxycholic acid supplementation did not affect body weight, food intake, and water intake. In females (A) Body weight. (B) Food intake. (C) Water intake. (D) Kidney weight/body weight ratio. (E) Heart weight/body weight ratio. (F) Liver weight/body weight ratio. (G) Spleen weight/body weight ratio. In males (H) Body weight. (I) Food intake. (J) Water intake. (K) Kidney weight/body weight ratio. (L) Heart weight/body weight ratio. (M) Liver weight/body weight ratio. (N) Spleen weight/body weight ratio. *Tgr5*: Takeda G-protein coupled receptor 5, GDCA: Glycodeoxycholic acid. n = 6-8 rats/group/sex. Unpaired, parametric two-tailed Student’s *t* test with a 95% confidence interval was used to compare data between groups. All data are mean ± SEM.

**Figure S8:** Glycodeoxycholic acid supplementation enhanced serum total bile acid pool and cholesterol level. In females (A) Total bile acids (TBA). (B) Total cholesterol. (C) Triglyceride. (D) Alanine aminotransaminase (ALT). (E) Aspartate aminotransferase (AST). (F) Alkaline phosphatase (ALP). In males (G) Total bile acids (TBA). (H) Total cholesterol. (I) Triglyceride. (J) Alanine aminotransaminase (ALT). (K) Aspartate aminotransferase (AST). (L) Alanine phosphatase (ALP). GDCA: Glycodeoxycholic acid. n = 5-8 rats/group/sex. Unpaired, parametric two-tailed Student’s *t* test with a 95% confidence interval was used to compare data between groups. All data are mean ± SEM, *p<0.05, **p<0.01.
